# Determining Chemical Reactivity Driving Biological Activity from SMILES Transformations: The Bonding Mechanism of Anti-HIV Pyrimidines 

**DOI:** 10.3390/molecules18089061

**Published:** 2013-07-30

**Authors:** Mihai V. Putz, Nicoleta A. Dudaş

**Affiliations:** Laboratory of Computational and Structural Physical Chemistry for Nanosciences and QSAR, Biology-Chemistry Department, West University of Timişoara, Pestalozzi Str. No. 16, Timişoara 300115, Romania; E-Mail: nicole_suceveanu@yahoo.com

**Keywords:** anti-HIV activity, 1,3-disubstituted uracil derivatives, QSAR, SMILES, electronegativity, chemical hardness, chemical power, electrophilicity, chemical reactivity principles, lipophilicity

## Abstract

Assessing the molecular mechanism of a chemical-biological interaction and bonding stands as the ultimate goal of any modern quantitative structure-activity relationship (QSAR) study. To this end the present work employs the main chemical reactivity structural descriptors (electronegativity, chemical hardness, chemical power, electrophilicity) to unfold the variational QSAR though their min-max correspondence principles as applied to the Simplified Molecular Input Line Entry System (SMILES) transformation of selected uracil derivatives with anti-HIV potential with the aim of establishing the main stages whereby the given compounds may inhibit HIV infection. The bonding can be completely described by explicitly considering by means of basic indices and chemical reactivity principles two forms of SMILES structures of the pyrimidines, the Longest SMILES Molecular Chain (LoSMoC) and the Branching SMILES (BraS), respectively, as the effective forms involved in the anti-HIV activity mechanism and according to the present work, also necessary intermediates in molecular pathways targeting/docking biological sites of interest.

## 1. Introduction

There is a tremendous current demand for new materials and substances for the betterment of life, applications, health and the environment, but new synthesis cannot sufficiently guarantee the sustainability of the new compounds. In a global effort to diminish the toxicological and adverse effects of the multi-scale interaction and fate of chemicals *in silico* (computational) methods appear more and more as a viable alternative and prerequisite for any experimental endeavor, *in vitro* first, and then moving on to the final *in vivo* tests. Accordingly, an intimate relationship between the structure of a compound, in physicochemical terms, and the manifested *reactivity* (in the chemical realm), *activity* (in the bio-/eco-/pharmaco-logical realm) and *functionality* (in the nano-toxicology and technology realm) should be computationally established as a “road map” of expectations, conditions of use, prediction and prevention. In this context, the computational mathematical and statistical algorithms for modeling the chemical-biological interaction of a compound with organisms have become known as quantitative structure-activity relationships (QSAR) methods have come to the forefront. Especially in the last decade, they have evolved towards a regulatory framework, able to jointly address a variety of areas such as:

- Toxicological dose (endpoint) response and risk for spatio-temporal multi-scale prediction [1,2];
- Assessment of metabolic genotoxicity and screening of chemicals with bioaccumulation potential [3,4];
- Modeling of nanomaterials [5], including the oxidative stress-potential [6] and the toxicity of nanoparticles [7];
- Food and organic chemicals’ safety by computational analysis [8,9,10];
- Computational toxicology [11];
- Complex algebraic (networks) as well as simple arithmetic physiological activity and toxicity [12,13];
- Quantifying the dynamics of environmental nutrients and contaminants [14] with a view toward nanochemistry [15] and nanomedicine [16];
- Integrative structure-property and structure-activity computational workflows [17];
- Interspecies toxicity analysis [18];
- Design of safe drugs by employing structural similarity and computing toxicity predictions [19,20];
- Guidance rules for the domain of applicability in QSAR approaches [21,22];
- Considering, in relation to molecular structure, the molecular topology and quantum chemical descriptors among the basic causes of the observed toxicological properties, reactivity (or aromaticity) and activities [23,24,25,26];
- The assessment of multilinear models for molecular classes and large sets of chemicals with environmental activity [27,28];
- Establishing hierarchical models for the human health effects of toxicants [29,30];
- The role of the hydrophobicity of new chemicals in relation with cells’ activity and associated mechanistic interactions [31,32].

Accordingly, in response to this increasing demand for benchmark principles to be followed by a reliable QSAR research project [33,34], the Organization for Economic Co-Operation and Development (OECD) had advanced a set of standard principles for the validation, for regulatory purposes, of (quantitative) structure-activity relationship models [35,36,37,38]. In short, these principles are:

- QSAR-1: a defined endpoint;
- QSAR-2: an unambiguous algorithm;
- QSAR-3: a defined domain of applicability;
- QSAR-4: appropriate measures of goodness-of–fit, robustness and predictivity;
- QSAR-5: a mechanistic interpretation, if possible.

At this point one should distinguish between two main directions in which a QSAR study may be conducted, namely:

- *Drug design oriented*, which is generated through extensive database screening [39,40], similarity and domain considerations [41,42], producing QSAR models which should be then validated by internal [43,44], external and read-across techniques [45] so that finally the molecules or molecular fragments predicted as most active or inhibitive depending on the endpoint target can be selected;
- *Mechanism oriented*, which consists mainly in the identification of the fundamental types of interaction that happen at the chemical-to-biological scale so that the structural properties of a compound constitute the causes that can be related to the manifest and recorded effects at a biological site [46,47,48,49,50,51];

In phenomenological terms, while the first direction is more related to technology and to the prescriptions for new synthesis, the second QSAR route is more on the scientific side due to the fundamental approach it involves; nevertheless, they both are related since after all, drug design is based on the desired or assumed mechanism of action specific to a given class of compounds, so knowing or revealing the mechanism of action for a given chemical-biological interaction only based on QSAR models remains as the first and probably the most important stage in drug design process itself. 

Then, one faces with the true challenge, namely how to extract from a single or from a collection of QSAR models the “first causes” of a chemical-biological interaction. Fortunately, one may rely on the (multi) linear form of QSAR models since, when considered in terms of physicochemical parameters with mechanistic interpretation at the nano-chemical scale, they provide just a manifestation of the quantum superposition principle [52]: while each structural parameter is associated with a given state or “chemical movement” specific to that state, their linear superposition combines into the macroscopic effect recorded as bio-/eco-/pharmaco-activity. Within this paradigm one has then the conceptual and computational freedom in establishing the “order” of the chemical states/movements toward the concerned endpoint. This direction has proven fruitful in assigning many useful QSAR tools thus enriching the related analysis and paving the way to *mechanistic drug design* through combination of various *in cerebro* (conceptual)—*in silico* (computational) approaches, such as:

- Considering the elements of a QSAR model, *i.e.*, both descriptors and activities as vectors in a multi-dimensional (chemical-biological) Banach-Hilbert (quantum) observable space [53,54,55,56];
- Considering the descriptors of a QSAR model mainly with observable or physicochemical character, e.g., hydrophobicity for cellular wall transduction (the translation motion), the total energy for steric optimization (rotation motion), polarizability for molecular cloud deformation (vibrational motion) [57,58], or more recently, through the chemical reactivity indices (electronegativity, chemical hardness and related quantities) for gaining more insight into the subtle bonding description (binding movement)—leading to the so called chemical reactivity driven biological activity picture (which will be used also in the present work) [59];
- Considering the systematical collection of QSAR models of descriptors in the previous entry along with their basic statistics, e.g., correlation factors, to be then employed either in an algebraic formulation of descriptor-activity correlations, proved to be always superior to the basic statistical one, or to entering in Euclidian paths among the computed endpoints [60], thus involving the square form of the correlation factor, to produce and compare minimum distances toward the most comprehensive (superior in correlation) QSAR model (in turn presumed to be the closest in the QSAR pool of models to the real/recorded activity). This approach, consecrated as Spectral-SAR [57,58,61,62,63], provides the mechanistic interpretation of biological action in terms of the hierarchy of structural causes (descriptors) along the least computed path across available QSAR mode;
- Considering, more recently, the way of improving the previous entry by extensive use of the variational approach in all stages of Spectral-SAR, from screening (*i.e*., selecting the training set) from a set of toxicants, to assessing the minimum path by considering the molecular passage through cellular walls accompanied by the partial chemical bonds in molecules [64], according with the Simplified Molecular-Input Line-Entry System (SMILES) [65,66,67,68].

This last point is from where the present work continues the idea of fully considering the SMILES structure in the computational development of QSARs, by calculating the associated descriptors and involving them in the mechanistic analysis. Actually, it was found that when using SMILES forms only for screening purposes, as in the present case for modeling the anti-HIV activity of selected uracil derivatives [69], the output mechanism provides an activated chemical-biological bonding not properly indicating the finalization of the ligand-receptor coupling to explain the anti-HIV activity. Therefore, the present report takes this concept one step further in order to complete the chemical-bonding picture by fully using the SMILES structures not only as a graphical tool but also considering them as an intermediate reality in the mechanistic picture of chemical ligand-biological receptor interaction yielding the recorded effect in the organism. To this end, the above mechanistic-oriented framework will be unfolded, by applying the OECD-QSAR principles to the present purpose and conceptual-computational stages [70], by combining Spectral-SAR methodology with variational principles of chemical reactivity driving biological activity and with the recursive minimization of paths across systematic QSAR with SMILES molecular (chemical reactivity) descriptors, to recognize the preferred hierarchy and the “first causes” that eventually result in the envisaged chemical binding and resulting anti-HIV activity. This mechanism may be further used in a subsequent stage when extensive validation and drug design studies to recognize the molecular shape and structure [71] which best accords with a particular mechanism of action can be envisaged.

## 2. Results and Discussion

### 2.1. OECD-QSAR Principle 1: A Defined Endpoint

According to OECD guidance, “the intent of QSAR Principle 1 (*defined endpoint*) is to ensure clarity in the endpoint being predicted by a given model, since a given endpoint could be determined by different experimental protocols and under different experimental conditions. It is therefore important to identify the experimental system that is being modeled by the (Q)SAR”. Note that the actual endpoint is still the inhibitory effect predicted by a series of 1,3-disubstituted uracil-based anti-HIV compounds [69] on reverse transcriptase [72,73,74,75] in highly active antiretroviral therapy (HAART) [76,77,78]. It arises, in principle, with the same binding mechanism as binding/breaking DNA, through a group of non-necessarily similar structures, giving rise to the following updating QSAR end-point approaches [79,80,81]:

- *(Eco-) toxicological studies*, having various end-points (such as inhibition, activation, death, sterility, irritations, *etc.*) yet produced by a group of similar molecules, *i.e.*, the case of *congeneric studies*;
- and *carcinogenic studies*, having essentially the same end-point as the exacerbated apoptosis that in principle diffuses in the organism no matter what the initial trigger point is, and may be initiated by highly structurally diverse molecules, being therefore classified as *non-congeneric studies*.

While the first case above is usually treated by ordinary (or direct) QSAR approaches, the second category is less frequently treated with the central QSAR dogma of congenericity. It therefore requires special approaches, such as the recently described residual-QSAR study [82]. This relies on the fact that if no direct high correlation can be found, then there is a high probability that the action is residual, complementary or indirect [83]. For this point one considers the working molecules under study the most likely form producing the considered end-point, namely the anti-HIV activity produced by uracil-based pyrimidines [69,84], along two aspects of their SMILES structure, as presented in Table 1:

- the longest SMILES molecular chain (LoSMoC), when bonds are breaking on aromatic rings and moieties such that the resulting molecule displays a sort of 2D form of the original molecule along the “fractalic” chain, assumed to be the first stage in intermediary molecular defolding targeting the receptor. The maximum SMILES chains in LoSMoC are presumably responsible for best transport/transduction of ligand molecules through cellular (lipidic) walls, after which they may be released with a modified structure due to their further ionization resulting from interactions with cellular layers; accordingly, another SMILES form is generated and considered next, namely:
- the Branching SMILES (BraS), representing the second phase of molecular defolding and providing ligand bond breakages such that many “bays” are formed, yet with consistent “arms” linking the short molecular “skeleton” aiming to favor the binding with a receptor in its pockets. Accordingly, the branching is not necessary in the same points of molecules through a series, but the maximum branching combined with equilibrium of branches is to be obtained in the final BraS. For instance, a long branch adjacent to a short one will not make a strong enough “float” to bind in a receptor pocket; therefore, the branching principle is to have the float-clefs balanced among themselves. To this end branching up to fourth order is performed for the molecules in Table 1.

However, one should note that the fact that the most drugs are ionized once immersed in a biological medium is in accordance with the present two-steps of SMILES conformations, since in each of them more nucleophilic compounds are considered due to the successive bonding breaking and the loss of pairs of electrons as the unfolding goes from the original to the LoSMoC to the BraS configuration. These SMILES metabolic intermediates acting as nucleophilic active sides are confirmed at least for fused and non-fused diazines [85], among which are also those based on pyrimidines, which have already demonstrated antiviral and anti-HIV activity [86,87,88,89] and antiinflammatory effects in general [90,91,92]. 

### 2.2. OECD-QSAR Principle 2: An Unambiguous Algorithm

According to the OECD guidance, the intent of QSAR-Principle 2 (unambiguous algorithm) is to ensure transparency in the predictive algorithm. In order to achieve this aim one needs reliable descriptors with physicochemical relevance. In this regard, the present QSAR modeling of ani-HIV activity employs the so called *chemical orthogonal space*–COS of chemical bonding [54,55], which is based on the main chemical reactivity indices and the principles of electronegativity (χ) and chemical hardness (η), alongside their related quantities such as chemical power index (π) and electrophilicity (ω) [93,94,95,96,97,98,99,100,101,102,103,104,105,106,107,108,109,110,111,112,113,114,115,116,117,118,119,120,121,122,123,124,125,126,127,128,129,130,131,132,133,134,135]. Their detailed description follows with the aim of better understanding the forthcoming QSAR- based mechanism of anti-HIV action for the present pool [136,137,138,139] of molecules.

#### 2.2.1. Electronegativity and Its Principles

Electronegativity is viewed as an instantaneous variation of total (or valence) energy for a neutral or charged system with *N*-electrons [93]:

(1) $$\chi \equiv -{\left(\frac{\partial E_{N}}{\partial N}\right)}_{V(r)}$$

It may be also be related to frontier electronic behavior by performing the central finite difference development of equation (1) in terms of ionization potential (IP) and electronic affinity (EA), thus facilitating further connection with the highest occupied and lowest unoccupied molecular orbitals, (HOMO and LUMO), respectively, according to Koopmans’ frozen spin orbitals’ theorem [94]:

(2) $$\chi_{FD}≅\frac{\left(E_{N_{0}-1}-E_{N_{0}})+(E_{N_{0}}-E_{N_{0}+1}\right)}{2}\equiv \frac{IP+EA}{2}≅-\frac{\varepsilon_{LUMO}+\varepsilon_{HOMO}}{2}$$

**Table 1:** Working molecules (IUPAC name and molecular weight MW are indicated ) and their corresponding SMILES topology, *i.e.* the longest SMILES molecular chain (LoSMoC) as upper entry and the Branching SMILES (BraS) as down entry, for each pyrimidine structure considered, along the common activity A = log_10_(1/EC_50_) employed from half maximal effective concentration (EC_50_, μM) antiviral activity of 1,3-disubstituted uracils against human immunodeficiency virus (HIV-1) [69], with AIDS code indicated [84], respectively. The solubility parameter of lipophilicity (LogP), and the chemical reactivity parameters such as electronegativity (χ) and chemical hardness (η), chemical power (π) and electrophilicity (ω) are considered within the semiempirical (AM1) framework (Polak-Ribiere conjugate gradient algorithm and geometry optimization till the root mean square RMS gradient was equal to or less than 0.01 kcal/Åmol) as provided by the Hyperchem 7.01 computational environment [140], while the chemical reactivity values were computed in terms of HOMO and LUMO from equations (14) and (15)—see text and Table 2, (7) and (10), respectively. SMILES legend is: 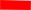 principal SMILES chain; 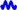 secondary SMILES branch; 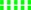 tertiary SMILES branch; 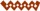 quaternary SMILES branch; = double bond; # triple bond; /,\ directional bonds; ( ) branch; C, N, F, S, I — atoms present in the molecule; c, n — atoms place in an aromatic ring; C_1/2/3_, N_1/2_, c_1/2/3_, n_2_ — connectivity points.

No.	Structure 2D	SMILES configurations		A	LogP	χ (eV)	η (eV)	π	ω (eV)
	IUPAC name MW AIDS code	LoSMoC	Code LoSMoC		... LoSMoC ...				
		BraS	Code BraS		... BraS ...				
**1**	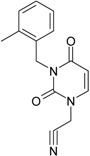 [3-(2-Methyl-benzyl)-2,4-dioxo-3,4-dihydro-2H-pyrimidin-1-yl]-acetonitrile 255.28 AIDS352092	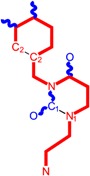	N#CCN1/C=C\C(=O)N(C1=O)Cc2ccc(C)c(C)c2	3.716698	0.91	23.107212	1.5817419	7.304356	168.78330
		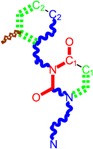	O=C1N(Cc(c(C)cc2)cc2)C(N(/C=C1\)CC#N)=O		0.44	13.240955	2.8324015	2.3374078	30.949511
**2**	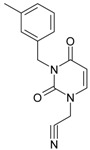 [3-(3-Methyl-benzyl)-2,4-dioxo-3,4-dihydro-2H-pyrimidin-1-yl]-acetonitrile 255.28 AIDS352093	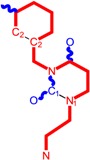	N#CCN1/C=C\C(=O)N(C1=O)Cc2cccc(C)c2	5.173925	0.47	22.812517	1.5937610	7.156819	163.26505
		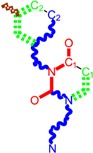	O=C1N(Cc(cc(C)c2)cc2)C(N(/C=C1\)CC#N)=O		0.44	13.043803	2.8273990	2.3066788	30.087865
**3**	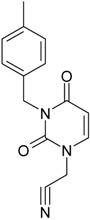 [3-(4-Methyl-benzyl)-2,4-dioxo-3,4-dihydro-2H-pyrimidin-1-yl]-acetonitrile 255.28 AIDS352094	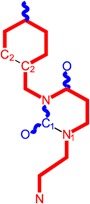	N#CCN1/C=C\C(=O)N(C1=O)Cc2ccc(C)cc2	4.023191	0.47	22.852718	1.5799314	7.232187	165.27512
		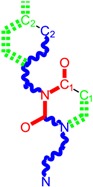	O=C1N(Cc(ccc2C)cc2)C(N(/C=C1\)CC#N)=O		0.88	13.149213	2.8323062	2.3212908	30.523148
**4**	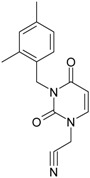 [3-(2,4-Dimethyl-benzyl)-2,4-dioxo-3,4-dihydro-2H-pyrimidin-1-yl]-acetonitrile 269.30 AIDS352888	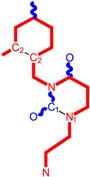	N#CCN1/C=C\C(=O)N(C1=O)Cc2ccc(C)cc2C	3.943095	1.06	22.695343	1.4889604	7.621204	172.96584
		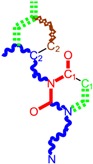	O=C1N(Cc2c(cc(cc2)C)C)C(N(/C=C1\)CC#N)=O		1.03	13.061603	2.7061581	2.4133112	31.521715
**5**	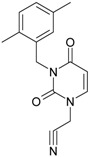 [3-(2,5-Dimethyl-benzyl)-2,4-dioxo-3,4-dihydro-2H-pyrimidin-1-yl]-acetonitrile 269.30 AIDS352889	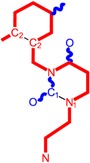	N#CCN1/C=C\C(=O)N(C1=O)Cc2cc(C)ccc2C	4.610833	1.06	22.961910	1.5967679	7.190121	165.09891
		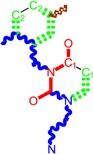	O=C1N(Cc(cc(C)c2)c(c2)C)C(N(/C=C1\)CC#N)=O		0.6	13.344068	2.8843065	2.3132194	30.867758
**6**	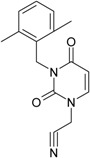 [3-(2,6-Dimethyl-benzyl)-2,4-dioxo-3,4-dihydro-2H-pyrimidin-1-yl]-acetonitrile 269.30 AIDS352890	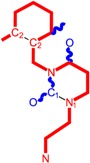	N#CCN1/C=C\C(=O)N(C1=O)Cc2c(C)cccc2C	3.707743	1.06	22.914792	1.5375402	7.45177	170.75577
		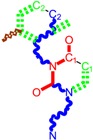	O=C1N(Cc(c(C)cc2)c(C)c2)C(N(/C=C1\)CC#N)=O		0.6	13.174123	2.7474378	2.3975289	31.585343
**7**	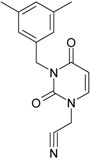 [3-(3,5-Dimethyl-benzyl)-2,4-dioxo-3,4-dihydro-2H-pyrimidin-1-yl]-acetonitrile 269.30 AIDS352095	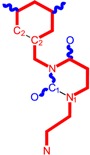	N#CCN1/C=C\C(=O)N(C1=O)Cc2cc(C)cc(C)c2	6.229147	0.63	22.322613	1.3441469	8.303636	185.35884
		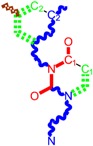	O=C1N(Cc(cc(C)c2)cc2C)C(N(/C=C1\)CC#N)=O		1.03	12.688503	2.5160717	2.5214906	31.993942
8	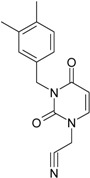 [3-(3,4-Dimethyl-benzyl)-2,4-dioxo-3,4-dihydro-2H-pyrimidin-1-yl]-acetonitrile 269.30 AIDS352891	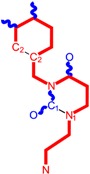	N#CCN1/C=C\C(=O)N(C1=O)Cc2ccc(C)c(C)c2	5.425968	0.63	22.513298	1.4966364	7.521298	169.32923
		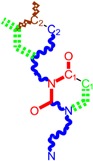	O=C1N(Cc(cc(c2C)C)cc2)C(N(/C=C1\)CC#N)=O		1.03	12.964034	2.7262701	2.3776137	30.823468
9	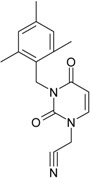 [3-(2,4,6-trimethyl-benzyl)- 2,4-dioxo-3,4-dihydro-2H-pyrimidin-1-yl]-acetonitrile 283.33 AIDS352892	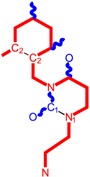	N#CCN1/C=C\C(=O)N(C1=O)Cc2c(C)cc(C)cc2C	3.716698	1.22	22.436637	1.3498377	8.310865	186.46785
		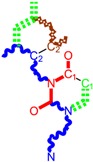	O=C1N(Cc2c(cc(cc2C)C)C)C(N(/C=C1\)CC#N)=O		1.62	12.848802	2.5836971	2.4865149	31.948740
**10**	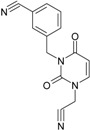 [3-(3-cyanophenyl)methyl-2,4-dioxo-3,4-dihydro-2H-pyrimidin-1-yl]-acetonitrile 266.26 AIDS352893	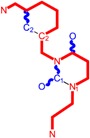	N#CCN1/C=C\C(=O)N(C1=O)Cc2cccc(c2)C#N	5.128427	0.04	22.981901	1.5807784	7.269172	167.05939
		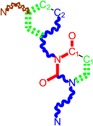	O=C1N(Cc(cc(C#N)c2)cc2)C(N(/C=C1\)CC#N)=O		0.01	12.984607	2.7188679	2.3878703	31.00556
**11**	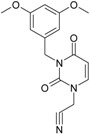 [3-(3,5-Dimethoxy-benzyl)-2,4-dioxo-3,4-dihydro-2H-pyrimidin-1-yl]-acetonitrile 301.30 AIDS352897	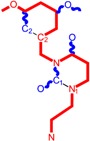	N#CCN1/C=C\C(=O)N(C1=O)Cc2cc(OC)cc(c2)OC	5.248720	-1.67	21.820275	1.0563595	10.32805	225.36097
		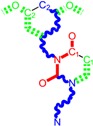	O=C1N(Cc(cc2OC)cc(OC)c2)C(N(/C=C1\)CC#N)=O		-0.72	12.366078	2.2360288	2.7651875	34.194524
**12**	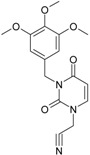 [3-(3,4,5-trimethoxy-benzyl)-2,4-dioxo-3,4-dihydro-2H-pyrimidin-1-yl]-acetonitrile 331.33 AIDS352898	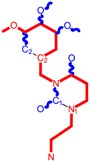	N#CCN1/C=C\C(=O)N(C1=O)Cc2cc(OC)c(OC)c(c2)OC	3.423658	-2.66	21.365171	1.0625102	10.0541	214.80760
		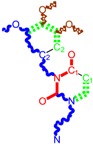	O=C1N(Cc2cc(c(OC)c(OC)c2)OC)C(N(/C=C1\)CC#N)=O		-2.26	12.143075	2.4593788	2.4687280	29.977950
**13**	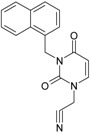	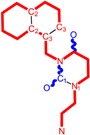	N#CCN1/C=C\C(=O)N(C1=O)Cc3c2ccccc2ccc3	5.268411	1.16	25.868615	1.4726275	8.78315	227.20792
	(3-Naphthalen-1-ylmethyl-2,4-dioxo-3,4-dihydro-2H-pyrimidin-1-yl)-acetonitrile 291.31 AIDS352899	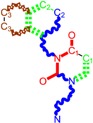	O=C1N(Cc(c(cc3)c(cc3)c2)cc2)C(N(/C=C1\)CC#N)=O		0.25	14.682316	2.7628433	2.6571026	39.012422
**14**	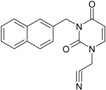 (3-Naphthalen-2-ylmethyl-2,4-dioxo-3,4-dihydro-2H-pyrimidin-1-yl)-acetonitrile 291.31 AIDS352900	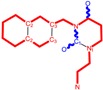	N#CCN1/C=C\C(=O)N(C1=O)Cc3cc2ccccc2cc3	4.435333	1.16	25.888824	1.3140309	9.850919	255.02871
		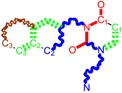	O=C1N(Cc(cc(ccc3)c2c3)cc2)C(N(/C=C1\)CC#N)=O		0.69	14.829177	2.6159392	2.8343888	42.031656
**15**	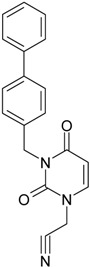 (3-Biphenyl-4-ylmethyl-2,4-dioxo-3,4-dihydro-2H-pyrimidin-1-yl)-acetonitrile 317.35 AIDS352901	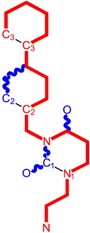	N#CCN1/C=C\C(=O)N(C1=O)Cc2ccc(cc2)c3ccccc3	4.236572	1.25	27.000458	1.2990428	10.39244	280.60074
		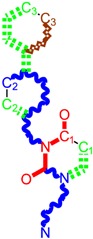	O=C1N(Cc(c2)ccc(c(cc3)ccc3)c2)C(N(/C=C1\)CC#N)=O		0.79	15.020930	2.3806514	3.1547941	47.387942
**16**	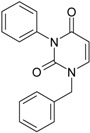 1-Benzyl-3-phenyl-1H-pyrimidine-2,4-dione 278.31 AIDS352902	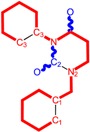	c1ccccc1CN2/C=C\C(=O)N(C2=O)c3ccccc3	3.665546	1.55	28.617336	1.4763650	9.691822	277.35413
		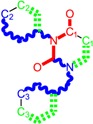	O=C1N(c(cc2)ccc2)C(N(/C=C1\)Cc(ccc3)cc3)=O		0.54	16.311764	2.7002385	3.0204302	49.268547
**17**	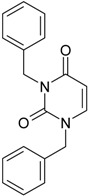 1,3-Dibenzyl-1H-pyrimidine-2,4-dione 292.34 AIDS352903	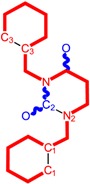	c1ccccc1CN2/C=C\C(=O)N(C2=O)Cc3ccccc3	4.954677	1.53	27.627131	1.4262804	9.685028	267.56953
		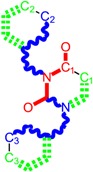	O=C1N(Cc(ccc2)cc2)C(N(/C=C1\)Cc(ccc3)cc3)=O		1.06	15.538736	2.6492805	2.9326332	45.569415
**18**	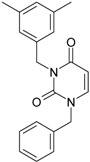 1-Benzyl-3-(3,5-dimethyl-benzyl)-1H-pyrimidine-2,4-dione 320.39 AIDS352096	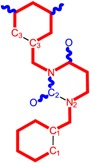	c1ccccc1CN2/C=C\C(=O)N(C2=O)Cc3cc(C)cc(C)c3	6.630784	1.84	25.860489	0.7302591	17.70638	457.89563
		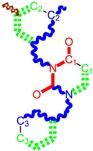	O=C1N(Cc(cc(C)c2)cc2C)C(N(/C=C1\)Cc(ccc3)cc3)=O		1.81	14.540931	1.5875011	4.5798175	66.594813
**19**	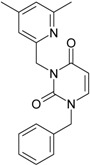 1-Benzyl-3-(4,6-dimethyl-pyridin-2-ylmethyl)-1H-pyrimidine-2,4-dione 321.38 AIDS352904	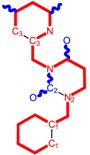	c1ccccc1CN2/C=C\C(=O)N(C2=O)Cc3nc(C)cc(C)c3	5.136082	0.41	26.114347	0.8253111	15.82091	413.15277
		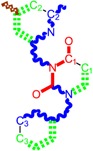	O=C1N(Cc(cc(C)c2)nc2C)C(N(/C=C1\)Cc(ccc3)cc3)=O		0.15	14.748792	1.7122755	4.3067812	63.519822
**20**	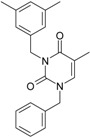 1-Benzyl-3-(3,5-dimethyl-benzyl)-5-methyl-1H-pyrimidine-2,4-dione334.42 AIDS352905	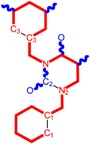	c1ccccc1CN2/C=C\(C)C(=O)N(C2=O)Cc3cc(C)cc(C)c3	5.841637	2.12	25.007275	1.0403700	12.01845	300.54873
		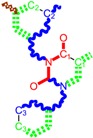	O=C1N(Cc(cc(C)c2)cc2C)C(N(/C=C1\C)Cc(ccc3)cc3)=O		2.39	14.063834	2.1272754	3.3055978	46.489379
**21**	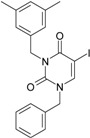 1-Benzyl-3-(3,5-dimethyl-benzyl)-5-iodo-1H-pyrimidine-2,4-dione 446.29 AIDS352906	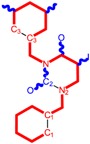	c1ccccc1CN2/C=C\(I)C(=O)N(C2=O)Cc3cc(C)cc(C)c3	4.379863	2.48	25.393186	0.8931783	14.21507	360.96592
		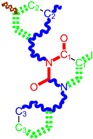	O=C1N(Cc(cc(C)c2)cc2C)C(N(/C=C1\I)Cc(ccc3)cc3)=O		2.53	13.656576	1.4894424	4.5844594	62.608023
**22**	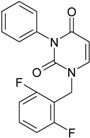 1-(2,6-Difluoro-benzyl)-3-phenyl-1H-pyrimidine-2,4-dione 314.29 AIDS352907	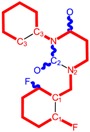	Fc1cccc(F)c1CN2/C=C\C(=O)N(C2=O)c3ccccc3	3.690369	1.08	28.610234	1.4786792	9.674253	276.78264
		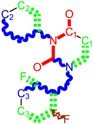	O=C1N(c(cc2)ccc2)C(N(/C=C1\)Cc(c(F)cc3)c(F)c3)=O		−0.66	16.175016	2.7665356	2.9233342	47.284980
**23**	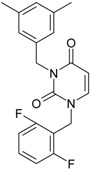 1-(2,6-Difluoro-benzyl)-3-(3,5-dimethyl-benzyl)-1H-pyrimidine-2,4-dione 356.37 AIDS352908	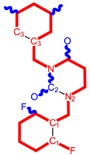	Fc1cccc(F)c1CN2/C=C\C(=O)N(C2=O)Cc3cc(C)cc(C)c3	6.939302	1.37	25.844444	0.7517152	17.19032	444.27415
		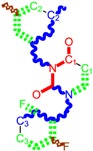	O=C1N(Cc(cc(C)c2)cc2C)C(N(/C=C1\)Cc(c(F)cc3)c(F)c3)=O		0.6	14.486247	1.5713578	4.6094680	66.773895
**24**	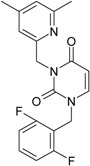 1-(2,6-Difluoro-benzyl)-3-(4,6-dimethyl-pyridin-2-ylmethyl)-1H-pyrimidine-2,4-dione357.36 AIDS352909	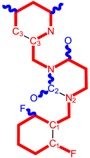	Fc1cccc(F)c1CN2/C=C\C(=O)N(C2=O)Cc3nc(C)cc(C)c3	5.193820	−0.06	26.085800	0.8406863	15.51458	404.71036
		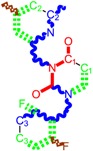	O=C1N(Cc(cc(C)c2)nc2C)C(N(/C=C1\)Cc(c(F)cc3)c(F)c3)=O		−1.05	14.690744	1.6779412	4.3776098	64.310348
**25**	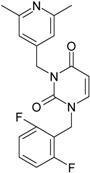 1-(2,6-Difluoro-benzyl)-3-(2,6-dimethyl-pyridin-4-ylmethyl)-1H-pyrimidine-2,4-dione 357.36 AIDS352910	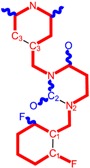	Fc1cccc(F)c1CN2/C=C\C(=O)N(C2=O)Cc3cc(C)nc(C)c3	3.886056	0.57	26.493803	0.9063530	14.61561	387.22308
		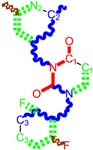	O=C1N(Cc(cc(C)n2)cc2C)C(N(/C=C1\)Cc(c(F)cc3)c(F)c3)=O		0.77	14.950333	1.7825743	4.1934669	62.693730
**26**	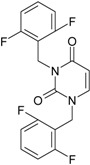 1,3-Bis-(2,6-difluoro-benzyl)-1H-pyrimidine-2,4-dione 364.30 AIDS352911	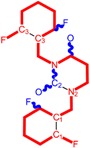	Fc1cccc(F)c1CN2/C=C\C(=O)N(C2=O)Cc3c(F)cccc3F	4.379863	0.59	27.958833	1.5546911	8.991764	251.39924
		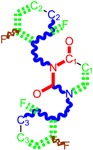	O=C1N(Cc(c(F)cc2)c(F)c2)C(N(/C=C1\)Cc(c(F)cc3)c(F)c3)=O		−1.34	15.611849	2.8690618	2.7207236	42.475527
**27**	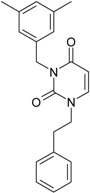 3-(3,5-Dimethyl-benzyl)-1-phenethyl-1H-pyrimidine-2,4-dione334.42 AIDS352912	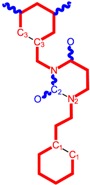	c1ccccc1CCN2/C=C\C(=O)N(C2=O)Cc3cc(C)cc(C)c3	5.206209	2.09	25.447501	0.8335692	15.26418	388.43520
		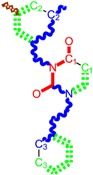	O=C1N(Cc(cc(C)c2)cc2C)C(N(/C=C1\)CCc(cccc3)c3)=O		2.06	14.410323	1.8477646	3.8993936	56.191522
**28**	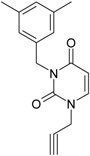 3-(3,5-Dimethyl-benzyl)-1-prop-2-ynyl-1H-pyrimidine-2,4-dione 268.32 AIDS352913	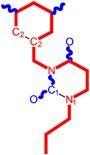	C#CCN1/C=C\C(=O)N(C1=O)Cc2cc(C)cc(C)c2	5.966576	0.77	21.628890	1.4603086	7.405589	160.17466
		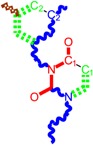	O=C1N(Cc(cc(C)c2)cc2C)C(N(/C=C1\)CC#C)=O		1.18	12.392809	2.5046350	2.4739751	30.659502
**29**	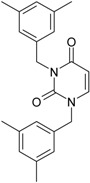 1,3-Bis-(3,5-dimethyl-benzyl)-1H-pyrimidine-2,4-dione348.44 AIDS352914	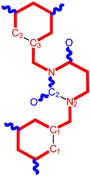	c1c(C)cc(C)cc1CN2/C=C\C(=O)N(C2=O)Cc3cc(C)cc(C)c3	6.283996	2.14	25.233546	0.8800182	14.33694	361.77196
		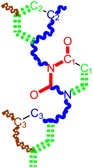	O=C1N(Cc(cc(C)c2)cc2C)C(N(/C=C1\)Cc(cc(cc3C)C)c3)=O		2.55	14.566107	1.9376961	3.7586149	54.748388
**30**	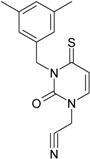 [3-(3,5-Dimethyl-benzyl)-2-oxo-4-thioxo-3,4-dihydro-2H-pyrimidin-1-yl]-acetonitrile 285.36 AIDS352915	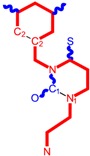	N#CCN1/C=C\C(=S)N(C1=O)Cc2cc(C)cc(C)c2	7.309803	1.28	21.897722	1.6182386	6.765913	148.15807
		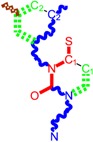	S=C1N(Cc(cc(C)c2)cc2C)C(N(/C=C1\)CC#N)=O		1.68	12.764862	3.0237637	2.1107572	26.943525
**31**	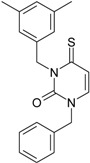 1-Benzyl-3-(3,5-dimethyl-benzyl)-4-thioxo-3,4-dihydro-1H-pyrimidin-2-one 336.45 AIDS352916	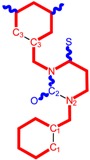	c1ccccc1CN2/C=C\C(=S)N(C2=O)Cc3cc(C)cc(C)c3	7.292429	2.49	25.217792	1.1471616	10.99139	277.17849
		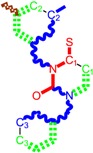	S=C1N(Cc(cc(C)c2)cc2C)C(N(/C=C1\)Cc(ccc3)cc3)=O		2.45	14.289267	2.4197012	2.9526925	42.191813
**32**	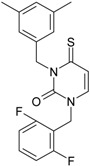 1-(2,6-Difluoro-benzyl)-3-(3,5-dimethyl-benzyl)-4-thioxo-3,4-dihydro-1H-pyrimidin-2-one 372.43 AIDS352917	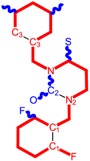	Fc1cccc(F)c1CN2/C=C\C(=S)N(C2=O)Cc3cc(C)cc(C)c3	7.229147	2.02	25.321304	1.0761564	11.76469	297.89740
		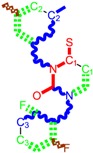	S=C1N(Cc(cc(C)c2)cc2C)C(N(/C=C1\)Cc(c(F)cc3)c(F)c3)=O		1.25	14.434969	2.3806265	3.0317586	43.763344

As such, in the course of a chemical reaction, or in chemical reactivity in general, electronegativity basically assures energetic stabilization through equalization of middle HOMO-LUMO levels among ligand (L) and receptor (R) active molecular structures; this is sustained by its inner definition [equation (1)] which identifies it with the negative of the chemical potential of a system, as according to Parr *et al.* [95], by the natural thermodynamic law of two fluids in contact its complex evolves towards equalization of the individual chemical potentials into a global one, while this principle, for the electronic fluid systems, was already consecrated from solid state physics [96], in chemistry it was coined by the so called *electronegativity equalization (EE) principle* ( Δχ=0 ), as originally stated by Sanderson under the assumption that “for molecules in their fundamental state, the electronegativities of different electronic regions in the molecule—are equal” [97]; however, its variational form was recently clarified within the context of the double variational procedure [98], specific to chemical systems:

(3) δχ≤0

under the minimum electronegativity principle stating that: “a chemical reaction is promoted so as to minimize further charge transfer between atoms-in-molecules or between molecular fragments within a complex” [99,100,101]. Nevertheless it was firstly formulated by Parr and Yang as under the maximum form favoring chemical reactivity [102,103]: “given two different sites with generally similar disposition for reacting with a given reagent, the reagent prefers the one which on the reagent’s approach is associated with the maximum response of the system’s electronegativity. In short, Δχ≥0 is good for reactivity (n. a.)”. Yet, for assessing the chemical stability the reverse form of the latter idea will be considered, from where the minimum electronegativity principle Δχ≤0 immediately results. However, in order to not conflict with the equality of electronegativity, this principle should be seen as a quantum fluctuation remnant effects in system upon the EE was consumed, *i.e.*, it needs to be minimized so that the system reaches stable equilibrium [104]. 

#### 2.2.2. Chemical Hardness and Its Principles

Chemical hardness is viewed as the instantaneous electronegativity change with charge [105]:

(4) $$\eta \equiv -\frac{1}{2}{\left(\frac{\partial \chi }{\partial N}\right)}_{V(r)}$$

It also supports the Koopmans’ frozen spin orbitals reformulation at the level of molecular frontier, *i.e.*, there where chemical reactivity takes place, through the expression [106]:

(5) $$\eta_{FD}=\frac{1}{2}{\left(\frac{\partial^{2}E_{N}}{\partial N^{2}}\right)}_{V(r)}≅\frac{E_{N_{0}+1}-2E_{N_{0}}+E_{N_{0}-1}}{2}=\frac{IP-EA}{2}≅\frac{\varepsilon_{LUMO}-\varepsilon_{HOMO}}{2}$$

At this point, while comparing Equations (2) and (5), it is clear that the electronegativity and chemical hardness may be viewed as the basis for an orthogonal space {χ,η|χ⊥η} for chemical reactivity analysis since the conceptual and practical differences noted between the energetic level characterizing the “experimental” electronegativity and the energetic gap characterizing the “experimental” chemical hardness, respectively [107,108].

Like electronegativity, chemical hardness also supports two types of equations accompanying the chemical reactions and transformations. The first one promoting equalization of chemical hardness Δη=0 of the atoms in a molecule or between molecular fragments in a complex or between adducts in a chemical bond refers to the so called *the hard and soft acids and bases (HSAB) principle* [109,110,111]; it was initially formulated by Pearson and says that “the species with a high chemical hardness prefer the coordination with species that are high in their chemical hardness, and the species with low softness (the inverse of the chemical hardness) will prefer reactions with species that are low in their softness, respectively” [112]. This leads to numerous applications in both inorganic and organic chemistry, since it practically reshapes the basic Lewis and Brönsted qualitative theories of acids and bases [113] into a rigorous orbital-based rule of chemical reactivity and bonding quantification. Nevertheless, being of a quantum nature, chemical hardness inherently contains fluctuations leading to the inequality or variational form of its evolution towards bonding stabilization; as such, within the abovementioned double-variational variational formalism the actual *maximum hardness principle* is advanced [114,115,116]:

(6) δη≥0

stating that the charge transfer during a chemical reaction or binding continues until the resulted bonded complex acquires maximum stability through hardness; *i.e.*, maximizing the HOMO-LUMO energetic gap thus impeding further electronic transitions [117]. It was originally based on the Pearson observation according which “there seems to be a rule of nature that molecules (or the many-electronic systems in general; n. a.) arrange themselves (in their ground or valence states; n. a.) to be as hard as possible” [113]; it also leads to the practical application merely through its inverse formulation; the chemical softness is in turn related with the polarizability features of a system; *i.e.*, as an observable quantity rooted in the quantum structure of the system; so that the minimum polarization principle was actually tested for various chemical systems [118]; e.g., to rotational barriers accounting for conformational properties and thus with the steric effects [119]; such that the actual chemical hardness variational principle of equation (6) is also indirectly validated.

#### 2.2.3. Chemical Power and Its Principle

Since noting the opposition of electronegativity and chemical hardness, *i.e.*, being the former associated with the tendency of the system to attract electrons and the latter with the tendency to inhibit the coordination and with the system stability, one may introduce the concept of *chemical power*, as the *dynamic charge* of atoms in a molecule, between molecular fragments or between adducts in a chemical bond, through the basic definition [59]:

(7) $$\pi =\frac{\chi }{2\eta }$$

**Figure 1:**
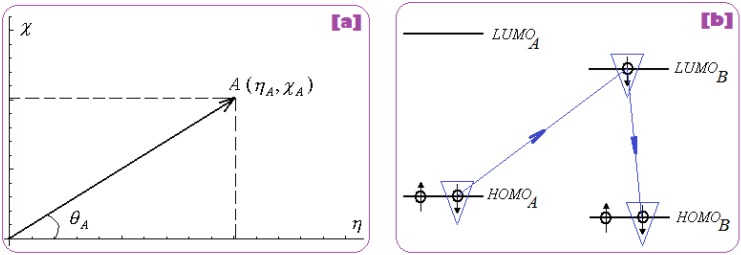
(**a**) Orthogonal hardness-electronegativity (*η* *–* *χ*) representation for an electronic system with coordinate A (*η_A_*, *χ_A_*); (**b**) the “ABB” mechanism of frontier chemical reactivity driven by chemical power in A-B bonding complex.

Initially, expression (7) was recognized as maximum electronic uptake in a bonding [120], yet one actually realizes that it gives us a sort of “reduced” or “normalized” electronegativity when its inertial hardness also counts. Moreover, for establishing a quantitative meaning one considers the Cartesian system where the coordinates are the hardness (on abscise) and electronegativity (on ordinate), see Figure 1a; in this framework there follows that:

(8) $$\pi =\frac{1}{2}\frac{\chi_{A}}{\eta_{B}}=\frac{1}{2}\tan(\theta_{A})≅-\Delta N_{A}$$

The last identity in (8) follows from chemical hardness-to-electronegativity definition (4) and allows the practical interpretation of chemical power in the chemical reactivity and bonding realm, providing the electronic charge transfer released by the adduct “A” when in bonding in an “A-B” complex, see Figure 1b.

Accordingly, the original frontier orbital HOMO_A_ is minimized to the HOMO_B_ in bonding, through the intermediate LUMO_B_. In variational terms, the chemical power index is associated with minimizing HOMOs in bonding by means of *charge transfer without spin changing*:

(9) δπ≤0

While principle (9) is consistent with principles relating minimum electronegativity and inverse of maximum of chemical hardness, it also emphasizes the necessity of the double variational principle when combined with Equation (8), *i.e.*, the released charge transfer of A in bonding is minimized so as to fit with the HOMO of bonding; in other terms, LUMO/HOMO_A_ and LUMO/HOMO_B_ levels also tend to equalize in bonding thus jointly fulfilling the conditions of equalization of electronegativity and chemical hardness. 

#### 2.2.4. Electrophilicity and Its Principle

Electrophilicity [120], further allows coupling of chemical power index with electronegativity to provide the energetic information of activation towards *charge tunneling* of the potential between adducts [59,64]:

(10) $$\omega =\chi \times \pi =\frac{\chi^{2}}{2\eta }$$

**Figure 2:**
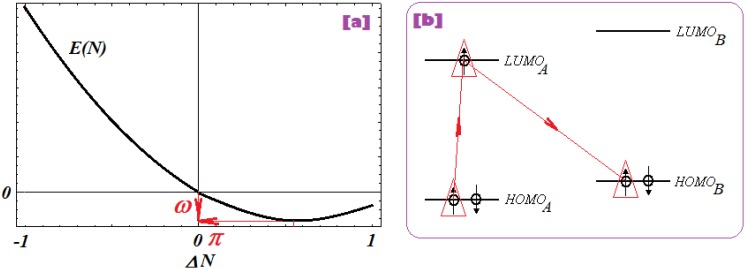
(**a**) Orthogonal representation of chemical power-electrophilicity (*π* *–* *ω*) scheme for a parabolic form of total energy respecting the number of electrons for an elementary reactivity (accepting-donating) range; (**b**) the “AAB” mechanism of frontier chemical reactivity driven by electrophilicity in a A-B bonding complex as the complementary/orthogonal one respecting the “ABB” counterpart of Figure 1(b).

Electrophilicity actually accounts for energy consumed by a system for manifesting its chemical power in a chemical orthogonal space see Figure 2a, essentially complementing it in bonding by electron transfer through tunneling between the bonding adducts, having the parent LUMO as an intermediate state, see Figure 2b as “orthogonal/complementary” to that of Figure 1b.

As a mixed reactive index electrophilicity was developed to characterize the electrophilic/ nucleophilic action of charge transfer through accepting/donating electrons, in modeling a variety of physical-chemical phenomena such as site selectivity [121,122], molecular vibrations and rotation [123], intramolecular and intermolecular reactivity patterns [124,125], solvent and external field effects [126,127,128] as well as biological activity and toxicity [59,64,129,130,131,132,133,134,135].

However, electrophilicity involves even stronger than the chemical power the *double* minimum character (through squaring of electronegativity and of its principle) which corresponds to charge penetration of the A-B energetic barrier towards fulfilling *electronic* *pairing* in a bonded complex. In practical circumstances, electrophilicity drives the electronic jump from HOMO_A_ to LUMO_A_ then relaxes to HOMO_B_ in an “A-B” bond complex thus covering the “AAB” pathway in chemical reactivity and bonding, see Figure 2b; in this case it minimizes the LUMO_B_-HOMO_B_ gap, as the inverse of chemical hardness as promoted by equation (10), that nevertheless leaves the bonding complex in an activated state which competes with minimization of electronegativity (through pairing) which tends to stabilizes the structure. For this reason the overall variational principle of electrophilicity assumes its minimization form:

(11) δω≤0

yet whether this is a characteristic of a reactive or stabilized bonding system remains an open issue and should be assessed for each case under study. 

These reactivity indices and principles are suited for analyzing the molecular interaction mechanism for a bonding complex chemically formed in a chemical-biological interaction, as is the present anti-HIV concerned action. 

### 2.3. OECD-QSAR Principle 3: A Defined Domain of Applicability

OECD guidance justifies the need to define an applicability domain (Principle 3) by the fact that (Q)SARs are reductionist models with inevitable limitations. These include limitations in terms of the types of chemical structures, physicochemical properties and mechanisms of action for which the models can generate reliable predictions [136,137,138,139]. 

This principle is inherently linked with the first OECD-QSAR endpoint criterion but equally influences the final mechanism of action, the “OECD-QSAR fifth commandment”. However, in the present anti-HIV study, establishing the domain of applicability is associated with the SMILES screening in searching of the working (trial) test of molecules, among the molecules of Table 1, as follows. 

The given chemical structures are employed via their highest occupied and lowest unoccupied molecular orbitals, HOMO and LUMO, respectively, to provide the basic chemical reactivity indices as such the electronegativity and chemical hardness, chemical power and electrophilicity, as previously described, since they are naturally interpreted in a successive and combined QSAR models by their associate chemical principles [59]. 

To accomplish such a goal, an original step was recently undertaken when for a QSAR series one considers also their counterpart Simplified Molecular Input Line Entry System (SMILES) transformations, which were assumed as being responsible for an intermediate stage in the molecular interaction mechanism targeting the receptor site [64]. However, the present endeavor continues the approach where the SMILES molecules were involved only in the screening for QSAR modeling, by effectively invoking also the SMILES structures in the QSAR models employed. This is because in the former stage the ligand-receptor binding mechanism remained unfinished at the level of activated 1,3-disubstituted uracil-reverse transcriptase complex, while the present ansatz is that the activated complex will be eventually relax and this can be studied by considering the computed structure parameters for the SMILES counterparts. Yet, as was pointed out in the previous paper [64], the mechanistic QSARs should be always driven and selected by the variational min-max principles, at all stages of conceptual and computational analysis. They will also be considered in the present analysis, having the additional SMILES molecular configurations as intermediates between the free molecules and the molecules binding to the biological receptor. 

Computationally, this behavior is reflected in considering the SMILES forms of Table 1 in ionization [+2n] states, with “n” representing the number of broken bonds in the gas-phase molecule. The computational framework chosen was the semiempirical AM1 as executed in the Hyperchem code [140], with which help the respective HOMO and LUMO states were determined, beyond the first order of frontier orbitals used in “custom” chemical reactivity calculations; see equations (2) and (5) for electronegativity and chemical hardness, respectively. This approach is also consistent with the “branching” effect at the energetic level of SMILES structures. Fortunately, within Koopmans’ approximation, such formulations exist up to the third order of compact finite differences and they look like [54,106,141]:

(12) $$\begin{array}{c} \chi_{CFD}=-\left[a_{1}\;\left(1-\alpha_{1}\;\right)\;+\frac{1}{2}b_{1}+\frac{1}{3}c_{1}\right]\frac{\varepsilon_{HOMO(1)}\;+\;\varepsilon_{LUMO(1)}}{2}\\ -\left[b_{1}+\frac{2}{3}c_{1}-2a_{1}\;\left(\alpha_{1}+\beta_{1}\right)\right]\frac{\varepsilon_{HOMO(2)}\;+\;\varepsilon_{LUMO(2)}}{4}\\ -\left(c_{1}-3a_{1}\beta_{1}\right)\;\frac{\varepsilon_{HOMO(3)}\;+\;\varepsilon_{LUMO(3)}}{6}, \end{array}$$

(13) $$\begin{array}{c} \eta_{CFD}=\left[a_{2}\left(1-\alpha_{2}+2\beta_{2}\right)\;+\frac{1}{4}b_{2}+\frac{1}{9}c_{2}\right]\frac{\varepsilon_{LUMO(1)}\;-\;\varepsilon_{HOMO(1)}}{2}\\ +\left[\frac{1}{2}b_{2}+\frac{2}{9}c_{2}+2a_{2}\;\left(\beta_{2}-\alpha_{2}\;\right)\right]\;\frac{\varepsilon_{LUMO(2)}\;-\;\varepsilon_{HOMO(2)}}{4}\\ +\left[\frac{1}{3}c_{2}-3a_{2}\beta_{2}\right]\frac{\varepsilon_{LUMO(3)}\;-\;\varepsilon_{HOMO(3)}}{6} \end{array}$$

When they are employed here under the spectral-like-resolution numerics [142], equations (12) and (13) reduce to the working ones [54,106,141]:

(14) $$\begin{array}{c} \chi_{CFD}^{SLR}=-1.06084\frac{\varepsilon_{HOMO(1)}\;+\;\varepsilon_{LUMO(1)}}{2}\\ +0.718869\frac{\varepsilon_{HOMO(2)}\;+\;\varepsilon_{LUMO(2)}}{4}\\ +0.31381\;\frac{\varepsilon_{HOMO(3)}\;+\;\varepsilon_{LUMO(3)}}{6}, \end{array}$$

(15) $$\begin{array}{c} \eta_{CFD}^{SLR}=0.582177\frac{\varepsilon_{LUMO(1)}\;-\;\varepsilon_{HOMO(1)}}{2}\\ +0.708161\frac{\varepsilon_{LUMO(2)}\;-\;\varepsilon_{HOMO(2)}}{4}\\ +0.022712\frac{\varepsilon_{LUMO(3)}\;-\;\varepsilon_{HOMO(3)}}{6} \end{array}$$

The analytical descriptors of equations (14) and (15) greatly help in considering the chain and branching modeling of actual molecules as being differentiated for LoSMoC and BraS intermediates also at the level of frontier chemical reactivity. As reported in Table 2:

- We consider only first orders of HOMO and LUMO for the LoSMoC molecules of Table 1;
- We consider all three orders of HOMO and LUMO for the BraS molecules of Table 1.

Values of χ & η, of Table 1 are based on the HOMO and LUMO entries of Table 2 combined with equations (14) and (15). They are further implemented in π & ω of equation (7) and (10) to provide the respective LoSMoC and BraS results in Table 1 as well. 

**Table 2:** The AM1 computed values (in electron-volts, eV) for the first three highest occupied and lowest unoccupied molecular orbitals in both variants as the longest SMILES molecular chain (LoSMoC, upper entry) and the Branching SMILES (BraS, lower entry), employed for computation of electronegativity (χ), chemical hardness (η), chemical power (π) and electrophilicity (ω), for the compounds of Table 1. Note that, in either LoSMoC or BraS forms, the overall compound was considered as carrying the [+2n] charge due to removed electronic pair out of each “broken bond” in SMILES configurations for compounds of Table 1. “X” indicates the truncation to the first order of HOMO and LUMO in LoSMoC calculations of electronegativity and chemical hardness of equations (14) and (15), respectively.

No.	HOMO1	LUMO1	HOMO2	LUMO2	HOMO3	LUMO3
	... LoSMoC ...					
	... BraS ...					
**1**	−24.49903	−19.06514	X	X	X	X
	−24.48801	−19.03451	−24.88237	−18.05411	−25.10602	−15.57611
**2**	−24.24188	−18.7667	X	X	X	X
	−24.23715	−18.75946	−24.69547	−17.93489	−24.84179	−15.32821
**3**	−24.25602	−18.82835	X	X	X	X
	−24.2567	−18.82977	−24.58191	−17.70927	−24.85183	−15.37954
**4**	−23.95141	−18.83626	X	X	X	X
	−23.95204	−18.83621	−24.28008	−17.60903	−24.88104	−15.38259
**5**	−24.38787	−18.90236	X	X	X	X
	−24.38787	−18.90236	−24.42277	−17.3528	−24.90982	−15.43411
**6**	−24.24172	−18.95968	X	X	X	X
	−24.23569	−18.95218	−24.49526	−17.86779	−25.04196	−15.49452
**7**	−23.35131	−18.73365	X	X	X	X
	−23.35188	−18.73767	−24.22514	−17.79723	−24.54057	−15.31291
**8**	−23.79299	−18.65147	X	X	X	X
	−23.79239	−18.65293	−24.13254	−17.38683	−24.7122	−15.20959
**9**	−23.46857	−18.83136	X	X	X	X
	−23.46979	−18.83921	−24.28395	−17.49789	−24.46192	−15.37871
**10**	−24.37925	−18.94867	X	X	X	X
	−24.38	−18.9506	−24.99142	−18.7636	−25.14861	−15.67584
**11**	−22.38345	−18.75445	X	X	X	X
	−22.38345	−18.75445	−23.79029	−17.31787	−24.21747	−15.29094
**12**	−21.96501	−18.31488	X	X	X	X
	−21.96844	−18.31149	−23.85501	−16.16856	−23.89945	−14.89846
**13**	−26.91465	−21.85561	X	X	X	X
	−26.91465	−21.85561	−27.7802	−20.69252	−28.33738	−18.9827
**14**	−26.66128	−22.14708	X	X	X	X
	−26.66128	−22.14708	−27.47999	−20.30796	−27.875	−19.37649
**15**	−27.68342	−23.22071	X	X	X	X
	−27.68553	−23.22033	−28.91865	−22.96564	−28.9519	−21.82943
**16**	−29.51216	−24.44028	X	X	X	X
	−29.52823	−24.42876	−29.75423	−23.06013	−30.9813	−22.86666
**17**	−28.49271	−23.59289	X	X	X	X
	−28.47523	−23.5581	−29.36592	−22.66404	−30.06262	−21.75203
**18**	−25.63183	−23.12311	X	X	X	X
	−25.62548	−23.11217	−26.88207	−22.20886	−27.55654	−20.01182
**19**	−26.0344	−23.19914	X	X	X	X
	−26.03953	−23.19181	−27.0533	−22.2293	−27.84065	−20.10159
**20**	−25.36022	−21.78615	X	X	X	X
	−25.36493	−21.78792	−26.68329	−20.71338	−27.06831	−19.37157
**21**	−25.47117	−22.40276	X	X	X	X
	−24.47218	−22.40179	−26.77381	−21.92655	−27.2391	−19.67952
**22**	−29.50944	−24.42961	X	X	X	X
	−29.5088	−24.42942	−30.31708	−23.21535	−30.95683	−22.84796
**23**	−25.65356	−23.07113	X	X	X	X
	−25.6511	−23.06654	−26.89824	−22.43524	−27.60502	−19.97251
**24**	−26.0339	−23.14582	X	X	X	X
	−26.03578	−23.15325	−27.02319	−22.45168	−27.88159	−20.08076
**25**	−26.5313	−23.41763	X	X	X	X
	−26.55279	−23.43647	−27.38203	−22.60093	−28.51525	−20.85345
**26**	−29.02596	−23.685	X	X	X	X
	−28.90689	−23.43443	−29.78576	−22.74551	−29.8298	−21.98785
**27**	−25.41998	−22.55635	X	X	X	X
	−25.42157	−22.55341	−26.66657	−21.11333	−27.34483	−19.49103
**28**	−22.8969	−17.88018	X	X	X	X
	−22.90148	−17.87531	−22.96332	−17.29096	−24.06666	−14.20252
**29**	−25.29808	−22.27488	X	X	X	X
	−25.30234	−22.27582	−25.91112	−20.09649	−26.56577	−19.35705
**30**	−23.42159	−17.86232	X	X	X	X
	−23.42258	−17.86581	−23.58074	−15.8222	−23.9511	−15.31823
**31**	−25.7421	−21.80116	X	X	X	X
	−25.74458	−21.80102	−27.02937	−20.01162	−27.54453	−19.79035
**32**	−25.71771	−22.0207	X	X	X	X
	−25.71681	−22.02284	−27.02077	−19.81276	−27.47808	−19.75182

**Figure 3:**
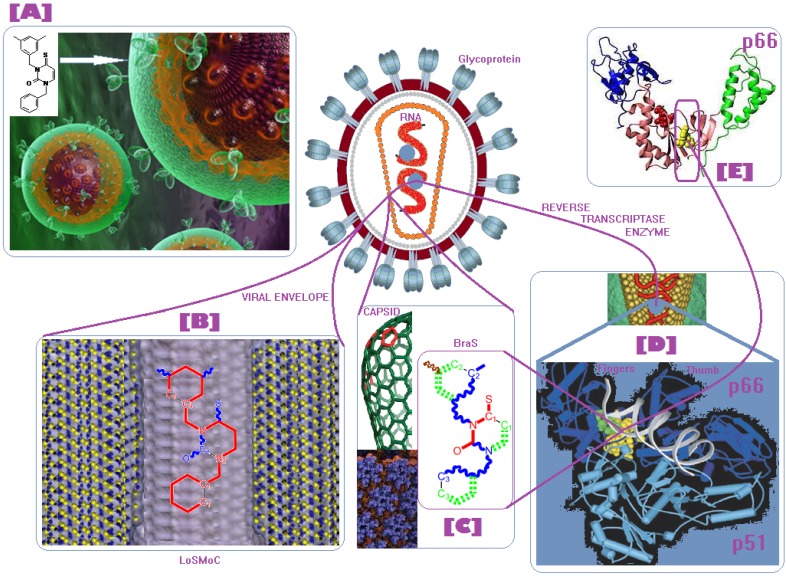
The mechanism of molecular interaction of the 1,3-disubstituted uracils, with prototype no. 31 of Table 1 (since belonging to all selected QSAR-SMILES criteria and cases of Table 3) against human immunodeficiency virus (HIV-1), after Ref. [143], through five stages: (**A**) the free molecular attack on the HIV viral envelope, after Ref. [144]; (**B**) the passage of the lipidic viral envelope of HIV under the form of longest SMILES molecular chain (LoSMoC) of Table 1, after Refs. [64,145,146]; (**C**) the transport though the protein layer of HIV capsid, after Refs. [147,148], yielding the Branching SMILES (BraS) configuration of Table 1 that further binds in (**D**) with the palm active region of p66 monomer of reverse transcriptase (RT), after Refs. [149,150], towards (**E**) the competitively inhibiting the RT by the formed ligand-receptor complex, after Ref. [151], by means of chemical reactivity frontier electronic transfer as detailed in the Figure 4.

Along with the different hydrophobicities for LoSMoC and BraS molecules, these chemical-physical descriptors are further employed by QSAR modeling to explain the chemical-biological binding of the actual series of pyrimidines to the reverse-transcriptase enzyme in HIV cells causing its inhibition for further action against the host organism’s cells. Actually, we have to explain by variational QSAR models how the anti-HIV mechanism of Figure 3 [143,144,145,146,147,148,149,150,151] is possible by means of SMILES chain and branching intermediates such as the LoSMoC and BraS conformations considered in Table 1 and based only on their chemical reactivity descriptors. 

**Table 3:** Case (i): screening based on SMILES central chain and Case (ii): screening based on SMILES central N-atom neighbors (N3 atom of the pyrimidine) for chain length and atomic neighboring in longest SMILES molecular chain (LoSMoC) in upper entry and the Branching SMILES (BraS) in down entry for various versions (V’s) of SMILES based screening criteria along the molecules of Table 1, respectively. The correlation factors are given for full dependency of parameters of Table 1, *i.e.*, A = A (χ, η, π, ω, logP), and for statistical error tolerance of 0.0001, unless otherwise indicated for the best correlation’s combination such that the Topliss-Costello rule [152] for ratio molecule-to-descriptors ≥ 4 to be generally respected (at least for χ & η as the main QSAR descriptors); the marked correlation corresponds with selected criteria and implicitly with the working molecular pool of Table 1 for each SMILES configuration (LoSMoC and BraS) and screening case (i and ii) further considered (see text).

Index	Criteria	CASE (i)		Case (ii)	
		Molecules	R_QSAR_	Molecules	R_QSAR_
**V1 LoSMoC**	Between 15–16 atoms LoSMoC	1–4, 6–11, 28	0.90371960 ^(a)^	1–9, 28	0.92402295 ^(c)^
***V1 BraS***	*Main chain and secondary branch with maximum 14 atoms*	*2–11, 13, 14, 16, 17, 22, 28*	*0.53158997*	*2, 3, 5–9, 13, 14, 16, 17, 22, 28, 29*	0.70384894
**V2 LoSMoC**	Between 18–21 atoms LoSMoC	13–17, 19, 21, 22, 24, 26, 31, 32	**0.75180080**	15–18, 21–23, 27, 29, 31, 32	0. 95150144 ^(b)^
***V2 BraS***	*Main chain and secondary branch with minimum 14 atoms*	*7, 11, 12, 15–17, 19, 22, 24–26, 28, 30–32*	*0.95109419*	*7, 15–17, 20, 21, 22, 27, 28, 29, 30–32*	***0.87354213***
**V3 LoSMoC**	At least one triple bond in the main chain LoSMoC	1–7, 9–11, 13, 14, 28, 30	0.56411064	1–4, 6, 7, 9, 13, 15, 28, 30	0.49202776
***V3 BraS***	*Secondary and tertiary branches with maximum 14 atoms*	*2–10, 13, 14, 28*	*0.62469181*	*1–7, 9, 13, 14, 28*	*0.75756597*
**V4 LoSMoC**	More than three branches in the main chain LoSMoC	2–4, 6–11, 19, 21, 22, 24–26, 28, 30–32	0.43357261	2–4, 6, 7, 9, 15,20–23, 27–32	0.61510478
***V4 BraS***	*Secondary and tertiary branches with minimum 14 atoms*	*11, 15–17, 19, 21–25, 31, 32*	*0.64694148*	*15–17, 20–23, 27, 29, 31, 32*	*0.94183439*
**V5 LoSMoC**	More than four branches in the main chain LoSMoC	7–9, 11, 19, 21, 24–26, 28, 30–32	0.47454364	7, 8, 20, 23, 27–32	0.71500251 ^(d)^
***V5 BraS***	*Minimum 3 tertiary branches*	*6, 11, 15–17, 19, 22–26, 31, 32*	***0.94899619***	*6, 15–17, 20–23,* *27, 29, 31, 32*	*0.64718879*
**V6 LoSMoC**	Ramifications of LoSMoC main chain containing groups formed only carbon and hydrogen atoms (except common = O, C = O)	2–4, 6–10, 19, 28, 30, 31	0.71050966 ^(b)^	2–4, 6, 7, 9, 15, 20,27–31	**0.64508095**
***V6 BraS***	*Minimum 1 quaternary branching*	*1, 2, 4, 6–8, 10,13–15, 19, 21–25, 28, 30–32*	*0.48549586*	*1, 2, 4, 6–8,13, 14, 20–23, 27–29, 30–32*	*0.63906586*
**V7 LoSMoC**	Ramifications of LoSMoC main chain containing groups consisting of a single atom or –CH_3_ groups (except common = O, C = O)	2–7, 9, 10, 19, 22,24–26, 28, 30–32	0.57636501	2–4, 6, 7, 9, 20–22,27– 32	0.61600596 ^(e)^
***V7 BraS***	*One of the secondary branches with minimum one triple bond*	*1–7, 9–11, 13–15, 28*	*0.63904635*	*1–7, 9, 13–15, 28*	*0.73556023 ^(d)^*
**V8 LoSMoC**	At least one branch for the last 6 points main chain LoSMoC	2–4, 6–11, 19, 23–25, 28, 30, 32	0. 51837657	2–4, 6, 7, 9, 20, 21, 27–32	0.69314160 ^(d)^
***V8 BraS***	*The secondary branch linked with C2 of pyrimidinic nucleus with minimum 2 heteroatoms*	*1–* *6, 8–11, 13–* *15*	*0.58368204*	*1–6, 8, 9, 13–15*	*0.57765388 ^(f)^*
**V9 LoSMoC**	LoSMoC main chain contains after N3 atom of the pyrimidine nucleus (central main chain LoSMoC) a group –CH_2_–	1–7, 9–11, 13–15, 19, 21, 24–26, 28, 30–32	0.37650771	1–8, 13–15, 20, 21, 27–32	0.63047473
***V9 BraS***	*The secondary branch linked with N3 of pyrimidinic nucleus contains only C and H atoms*	*1–* *8, 10, 11, 13–17, 25, 26, 28, 30–32*	*0.63881109*	*1–8, 13–17, 20, 21, 27–29, 30–32*	*0.72514327*
***V10 BraS***	*The secondary branch linked with N3 of pyrimidinic nucleus contains 4 Carbon atoms*	*2–* *4, 6 8–* *10, 13, 14, 16, 19, 22, 24, 26*	*0.61480396*	*2–6, 8, 9, 13, 14, 16, 17, 22*	*0.53480139*
***V11 BraS***	*The secondary branch linked with N3 of pyrimidinic nucleus contains 5–6 Carbon atoms*	*7, 12, 15, 18, 21, 23, 25, 28, 30–32*	*0.66627959*	*7, 15, 18, 20, 21, 23, 28, 29, 30–32*	*0.59914507*
***V12 BraS***	*The tertiary branching are formed by maximum 3 atoms of C and H*	*2, 4–10, 13, 16, 19, 21–25, 28, 30–32*	*0.38470862*	*2, 4, 6–9, 13, 16–18, 20, 21, 28–32*	*0.61909773*
***V13 BraS***	*The tertiary branches are formed only by C and H atoms*	*2–* *10, 13, 14, 16, 17, 19, 28, 30, 31*	*0.56415743*	*2–9, 13–16, 20, 27–31*	*0.64691170*
***V14 BraS***	*Quaternary branching are contains only one C atom or CH3 group*	*1, 2, 5–7, 21–25, 28, 30–32*	*0.57731047*	*2, 5, 6, 20–23, 27, 28, 30–32*	*0.72850903*
***V15 BraS***	*A single quaternary branching with maximum 2 atoms (C/O) and H*	*1, 2, 5–* *7, 19, 21, 22, 28, 30, 31*	*0.93051865*	*1, 2, 5, 6, 20–22,* *27, 28, 30, 31*	*0.90565106*

(a) A = A(χ, η, ω, logP); (b) within statistical error tolerance 0.00002; (c) within statistical error tolerance 1E^−25^; (d) within statistical error tolerance 0.00004; (e) within statistical error tolerance 0.00008; (f) within statistical error tolerance 0.00003;

QSAR analysis requires a preliminary screening such that out of the available pool of molecules the ones that further fulfill certain similarity criteria with an increased degree of correlation are retained. 

This stage is presented in Table 3 separately for LoSMoC and BraS and for each such molecular defolding, and separately for the SMILES central chain case (i) as well as for the N3- pyrimidine atom neighbors case (ii) due to its central role in obtaining the spiroheterocyclic compounds and their reaction pathways [85], which are also presumably defolded in the chemical-biological interaction. Note that, consistent with the previous branching considerations the criteria for BraS are almost doubled with respect to the LoSMoC. The results of Table 3 leaves us with two sets of molecules for each SMILES intermediate, while they are not necessary selected based only upon the highest correlation factor recorded, but through a compromise between the correlation factor and the number of chemical reactivity variables and with the number of compounds employed in the correlation. As such, for each LoSMoC/BraS cases (i)/(ii) one should chose the molecular sets presenting the best combination between:

- higher correlation factors;
- screening correlations having maxima of variables as descriptors;
- almost equal sets of compounds producing the precedent points;
- sets of compounds fulfilling the Topliss-Costello rule [152], or at least respecting the basic/independent descriptors of electronegativity and chemical hardness plus the hydrophobicity measure.

This way, the selected LoSMoC cases’ variants are:

- the case (i)/V2 was chosen over V1 since it better fulfills the above criteria (*e.g*. being based on all variables and on 12 compounds and not on four variables and 11 compounds like V1);
- the case (ii)/V6 was chosen despite the fact versions V1 and V2 have lesser compounds in the set, and to be closer to the previous case, for molecular sets’ cardinals.

On similar grounds, the selected BraS cases’ variants are:

- the case (i)/V5 over variant V2 since it has a minimum of three tertiary branching instances, while being in the similar correlation range, so that it better fulfills the “spirit” of molecular branching;
- the case (ii)/V2 over versions V4 and V15 (with lesser compounds in the set), being nevertheless in the same range of higher correlations and having the same cardinal of molecules in the set as its companion case (i)/V5

They are further used for integration in appropriate measures towards establishing the anti-HIV mechanism of action.

### 2.4. OECD-QSAR Principle 4: Appropriate Measures of Goodness-of-Fit, Robustness and Predictivity

OECD-QSAR principle 4 makes a distinction between the *internal performance of a model* as represented by goodness-of-fit and robustness or the correlation within the trial set of molecules and *the predictivity of a model* as determined by external validation on a test set of molecules [153,154].

However, in the present work we are considering internal measures of the present QSAR models (unfolded in Table 4) by their minimal search–formally written as:

(16) $$\delta \left[Y_{I},Y_{II},...,Y_{V}\right]=0$$

where $Y_{I},Y_{II},...,Y_{V}$ are the actual various computed endpoints, by means of the Euclidean paths across the available QSAR models, according with the rule [64]:

(17) $$\delta {\left\{|Y_{Ii}\rangle ,|Y_{IIi}\rangle ,|Y_{IIIi}\rangle ,|Y_{V}\rangle \right\}}_{i-PATH}={\left\{\sum_{\theta =I,II}{\left(R_{\theta i}-R_{(\theta +I)i}\right)}^{2}+{\left(R_{IIIi}-R_{Vi}\right)}^{2}\right\}}_{i-PATH}^{1/2}$$

with the results presented in Table 5.

**Table 4:** Statistical correlation results obtained for cases V2/(i) and V6/(ii) for longest SMILES molecular chain (LoSMoC) and respectively for the cases V5/(i) and V2/(ii) for branching SMILES (BraS) selected compounds’ sets form Table 3 with respective molecules of Table 1 (detailed respective QSAR models dependencies on chemical reactivity parameters are provided in Supplementary Material—Table S1).

No.	A(x)	LoSMoC		*BraS*	
		R_Case V2/(i)_	R_Case V6/(ii)_	*R_Case V5/(i)_*	*R_Case V2/(ii)_*
**I_1_**	A(logP)	0.36160241	0.43043863	*0.45645057*	*0.51687516*
**I_2_**	A(χ)	0.70875308	0.04142206	*0.32832072*	*0.63329686*
**I_3_**	A(η)	0.3850668	0.27082157	*0.3694801*	*0.10466918*
**I_4_**	A(π)	0.20001171	0.23419593	*0.23910446*	*0.36217604*
**I_5_**	A(ω)	0.0679732	0.21014	*0.12316764*	*0.52996859*
**II_1_**	A(logP, χ)	0.72462236	0.54711991	*0.54563771*	*0.68322871*
**II_2_**	A(logP, η)	0.53462981	0.45498598	*0.58822038*	*0.78078563*
**II_3_**	A(logP, π)	0.4587341	0.47447182	*0.53086816*	*0.8624387*
**II_4_**	A(logP, ω )	0.40635079	0.49281211	*0.48406183*	*0.85830581*
**II_5_**	A(χ, η)	0.72042921	0.34882836	*0.44147923*	*0.65793015*
**II_6_**	A(χ, π)	0.72662887	0.32861178	*0.42540934*	*0.67176394*
**II_7_**	A(χ, ω)	0.72663277	0.33323936	*0.41607475*	*0.67165975*
**II_8_**	A(η, π)	0.74023092	0.31232276	*0.46816571*	*0.69205634*
**II_9_**	A(η, ω)	0.74918964	0.3278778	*0.47282745*	*0.6980058*
**II_10_**	A(π, ω)	0.72422189	0.31072122	*0.4687647*	*0.66987725*
**III_1_**	A(logP, χ, η )	0.72946153	0.54741756	*0.62478127*	*0.83591477*
**III_2_**	A(logP, χ, π)	0.73229267	0.54735654	*0.62197159*	*0.86508134*
**III_3_**	A(logP, χ, ω)	0.73214282	0.5471543	*0.61493693*	*0.86624574*
**III_4_**	A(logP, η, π)	0.74609564	0.48854915	*0.62416978*	*0.87096819*
**III_5_**	A(logP, η, ω )	0.751297	0.51239927	*0.63374038*	*0.86007179*
**III_6_**	A(logP, π, ω)	0.72648755	0.52806785	*0.65025857*	*0.86552207*
**III_7_**	A(χ, η, π)	0.75053661	0.35028746	*0.4752325*	*0.7019648*
**III_8_**	A(χ, η, ω)	0.74939285	0.34885082	*0.52544907*	*0.70077495*
**III_9_**	A(χ, π, ω )	0.72663285	0.35789332	*0.83429197*	*0.67179626*
**III_10_**	A(η, π, ω)	0.74919138	0.33193549	*0.47362344*	*0.70165085*
**V**	A(logP, χ, η, π, ω)	0.7518008	0.64508095	*0.94899619*	*0.87354213*

**Table 5:** *Endpoint paths* and their lengths (δ) considered for the best/relevant QSAR’s correlations’ models of Table 4, in cases V2/(i) and V6/(ii) for longest SMILES molecular chain (LoSMoC) and in cases V5/(i) and V2/(ii) for branching SMILES (BraS) selected compounds’ sets from Table 3, upon the Euclidian metrics of Equation (17) applied on the first four shortest intermediary QSAR models of Table 4; the overall first three shortest path-lengths are identified in each configuration case by bolding and labeling as alpha (α), beta (β) and gamma (γ) superscripts, respectively.

LoSMoC				*BraS*			
Path	δ_V2/(i)_	Path	δ_V6/(ii)_	*Path*	*δ_V5/(i)_*	*Path*	*δ_V2/(ii)_*
I1-II1-III5-V	0.363999003	I1-II1-III1-V	0.15216027	*I1-II1-III5-V*	*0.339267818*	*I1-II1-III3-V*	*0.247430746*
I1-II1-III7-V	0.363945917	I1-II1-III2-V	0.15219933	***I1-II1-III6-V***	**0.328852605 ^γ^**	*I1-II1-III4-V*	*0.250851034*
I1-II1-III8-V	0.363872037	I1-II1-III3-V	0.15232909	***I1-II1-III9-V***	**0.323160465 ^β^**	*I1-II1-III6-V*	*0.246918396*
I1-II7-III5-V	0.36586301	I1-II2-III1-V	0.13669055	*I1-II2-III5-V*	*0.344705061*	*I1-II2-III3-V*	*0.277498475*
I1-II7-III7-V	0.365814373	I1-II2-III2-V	0.13669292	*I1-II2-III6-V*	*0.332349493*	*I1-II2-III4-V*	*0.27890546*
I1-II7-III8-V	0.365747157	I1-II2-III3-V	0.13670114	***I1-II2-III9-V***	**0.301780663 ^α^**	*I1-II2-III6-V*	*0.277296451*
I1-II8-III5-V	0.378790523	I1-II3-III1-V	0.12960764	*I1-II3-III5-V*	*0.339863056*	*I1-II3-III3-V*	*0.345661527*
I1-II8-III7-V	0.378770846	I1-II3-III2-V	0.12961931	*I1-II3-III6-V*	*0.330206319*	*I1-II3-III4-V*	*0.345678373*
I1-II8-III8-V	0.378746997	I1-II3-III3-V	0.12965837	*I1-II3-III9-V*	*0.332807819*	*I1-II3-III6-V*	*0.345670347*
I1-II9-III5-V	0.387593286	**I1-II4-III1-V**	**0.12810286 ^α^**	*I1-II4-III5-V*	*0.350074672*	*I1-II4-III3-V*	*0.341600891*
I1-II9-III7-V	0.387591632	**I1-II4-III2-V**	**0.1281234 ^β^**	*I1-II4-III6-V*	*0.342969246*	*I1-II4-III4-V*	*0.341675065*
I1-II9-III8-V	0.387594763	**I1-II4-III3-V**	**0.12819186 ^γ^**	*I1-II4-III9-V*	*0.369568114*	*I1-II4-III6-V*	*0.34160106*
I2-II1-III5-V	0.031042298	I2-II1-III1-V	0.51504227	*I2-II1-III5-V*	*0.392905816*	*I2-II1-III3-V*	*0.189846412*
I2-II1-III7-V	0.030413493	I2-II1-III2-V	0.51505381	*I2-II1-III6-V*	*0.383948387*	*I2-II1-III4-V*	*0.194283111*
**I2-II1-III8-V**	**0.029516257 ^β^**	I2-II1-III3-V	0.51509217	*I2-II1-III9-V*	*0.379084442*	*I2-II1-III6-V*	*0.18917817*
I2-II7-III5-V	0.030467382	I2-II2-III1-V	0.43487567	*I2-II2-III5-V*	*0.411103551*	***I2-II2-III3-V***	**0.170615371 ^β^**
**I2-II7-III7-V**	**0.029877668 ^γ^**	I2-II2-III2-V	0.43487642	*I2-II2-III6-V*	*0.40080012*	***I2-II2-III4-V***	**0.172894351 ^γ^**
**I2-II7-III8-V**	**0.029043119 ^α^**	I2-II2-III3-V	0.434879	*I2-II2-III9-V*	*0.37584055*	***I2-II2-III6-V***	**0.17028659 ^α^**
I2-II8-III5-V	0.033370142	I2-II3-III1-V	0.44987922	*I2-II3-III5-V*	*0.388579959*	*I2-II3-III3-V*	*0.229289585*
I2-II8-III7-V	0.033146038	I2-II3-III2-V	0.44988258	*I2-II3-III6-V*	*0.38016273*	*I2-II3-III4-V*	*0.22931498*
I2-II8-III8-V	0.032872383	I2-II3-III3-V	0.44989384	*I2-II3-III9-V*	*0.382424544*	*I2-II3-III6-V*	*0.229302881*
I2-II9-III5-V	0.04049457	I2-II4-III1-V	0.46505147	*I2-II4-III5-V*	*0.382158589*	*I2-II4-III3-V*	*0.225267191*
I2-II9-III7-V	0.040478734	I2-II4-III2-V	0.46505713	*I2-II4-III6-V*	*0.375660505*	*I2-II4-III4-V*	*0.225379654*
I2-II9-III8-V	0.040508702	I2-II4-III3-V	0.46507599	*I2-II4-III9-V*	*0.400091867*	*I2-II4-III6-V*	*0.225267448*
I3-II1-III5-V	0.340602068	I3-II1-III1-V	0.29305119	*I3-II1-III5-V*	*0.371725449*	*I3-II1-III3-V*	*0.606860446*
I3-II1-III7-V	0.340545335	I3-II1-III2-V	0.29307147	*I3-II1-III6-V*	*0.36224466*	*I3-II1-III4-V*	*0.608262992*
I3-II1-III8-V	0.340466377	I3-II1-III3-V	0.29313888	*I3-II1-III9-V*	*0.357085205*	*I3-II1-III6-V*	*0.606651729*
I3-II7-III5-V	0.342455676	I3-II2-III1-V	0.22803128	*I3-II2-III5-V*	*0.386400836*	*I3-II2-III3-V*	*0.681535121*
I3-II7-III7-V	0.342403714	I3-II2-III2-V	0.2280327	*I3-II2-III6-V*	*0.375420048*	*I3-II2-III4-V*	*0.682109209*
I3-II7-III8-V	0.342331902	I3-II2-III3-V	0.22803762	*I3-II2-III9-V*	*0.34864824*	*I3-II2-III6-V*	*0.681452889*
I3-II8-III5-V	0.355336832	I3-II3-III1-V	0.23734499	*I3-II3-III5-V*	*0.368802149*	*I3-II3-III3-V*	*0.75781421*
I3-II8-III7-V	0.355315856	I3-II3-III2-V	0.23735136	*I3-II3-III6-V*	*0.359922688*	*I3-II3-III4-V*	*0.757821894*
I3-II8-III8-V	0.355290432	I3-II3-III3-V	0.23737269	*I3-II3-III9-V*	*0.362310878*	*I3-II3-III6-V*	*0.757818233*
I3-II9-III5-V	0.364129287	I3-II4-III1-V	0.24859544	*I3-II4-III5-V*	*0.367313037*	*I3-II4-III3-V*	*0.753713772*
I3-II9-III7-V	0.364127526	I3-II4-III2-V	0.24860602	*I3-II4-III6-V*	*0.360547493*	*I3-II4-III4-V*	*0.753747392*
I3-II9-III8-V	0.364130859	I3-II4-III3-V	0.24864131	*I3-II4-III9-V*	*0.385936759*	*I3-II4-III6-V*	*0.753713849*
I4-II1-III5-V	0.525288611	I4-II1-III1-V	0.32781038	*I4-II1-III5-V*	*0.448453944*	*I4-II1-III3-V*	*0.369625875*
I4-II1-III7-V	0.525251826	I4-II1-III2-V	0.32782851	*I4-II1-III6-V*	*0.440627193*	*I4-II1-III4-V*	*0.371924125*
I4-II1-III8-V	0.525200637	I4-II1-III3-V	0.32788877	*I4-II1-III9-V*	*0.436395432*	*I4-II1-III6-V*	*0.369283099*
I4-II7-III5-V	0.527198557	I4-II2-III1-V	0.25851495	*I4-II2-III5-V*	*0.472588851*	*I4-II2-III3-V*	*0.42730628*
I4-II7-III7-V	0.527164806	I4-II2-III2-V	0.2585162	*I4-II2-III6-V*	*0.463653781*	*I4-II2-III4-V*	*0.428221331*
I4-II7-III8-V	0.527118165	I4-II2-III3-V	0.25852055	*I4-II2-III9-V*	*0.442255821*	*I4-II2-III6-V*	*0.42717511*
I4-II8-III5-V	0.540332774	I4-II3-III1-V	0.26942851	*I4-II3-III5-V*	*0.441695569*	*I4-II3-III3-V*	*0.500330351*
I4-II8-III7-V	0.54031898	I4-II3-III2-V	0.26943412	*I4-II3-III6-V*	*0.434308982*	*I4-II3-III4-V*	*0.500341989*
I4-II8-III8-V	0.540302262	I4-II3-III3-V	0.26945292	*I4-II3-III9-V*	*0.436290182*	*I4-II3-III6-V*	*0.500336444*
I4-II9-III5-V	0.549182204	I4-II4-III1-V	0.281784	*I4-II4-III5-V*	*0.426373085*	*I4-II4-III3-V*	*0.496246943*
I4-II9-III7-V	0.549181037	I4-II4-III2-V	0.28179334	*I4-II4-III6-V*	*0.420558718*	*I4-II4-III4-V*	*0.496298005*
I4-II9-III8-V	0.549183247	I4-II4-III3-V	0.28182447	*I4-II4-III9-V*	*0.44251816*	*I4-II4-III6-V*	*0.49624706*
I5-II1-III5-V	0.657190923	I5-II1-III1-V	0.3508471	*I5-II1-III5-V*	*0.534442949*	*I5-II1-III3-V*	*0.238824486*
I5-II1-III7-V	0.657161522	I5-II1-III2-V	0.35086404	*I5-II1-III6-V*	*0.52789265*	*I5-II1-III4-V*	*0.242366256*
I5-II1-III8-V	0.657120609	I5-II1-III3-V	0.35092035	*I5-II1-III9-V*	*0.524365617*	*I5-II1-III6-V*	*0.238293632*
I5-II7-III5-V	0.65912139	I5-II2-III1-V	0.27934081	*I5-II2-III5-V*	*0.563677521*	*I5-II2-III3-V*	*0.265077074*
I5-II7-III7-V	0.659094395	I5-II2-III2-V	0.27934197	*I5-II2-III6-V*	*0.556207653*	*I5-II2-III4-V*	*0.266549633*
I5-II7-III8-V	0.659057091	I5-II2-III3-V	0.27934599	*I5-II2-III9-V*	*0.53850008*	*I5-II2-III6-V*	*0.264865576*
I5-II8-III5-V	0.672348982	I5-II3-III1-V	0.29108509	*I5-II3-III5-V*	*0.52553652*	*I5-II3-III3-V*	*0.332571954*
I5-II8-III7-V	0.672337897	I5-II3-III2-V	0.29109028	*I5-II3-III6-V*	*0.519343768*	*I5-II3-III4-V*	*0.332589464*
I5-II8-III8-V	0.672324461	I5-II3-III3-V	0.29110768	*I5-II3-III9-V*	*0.521001709*	*I5-II3-III6-V*	*0.332581122*
I5-II9-III5-V	0.681219886	I5-II4-III1-V	0.30401219	*I5-II4-III5-V*	*0.502030388*	*I5-II4-III3-V*	*0.328514246*
I5-II9-III7-V	0.681218945	I5-II4-III2-V	0.30402085	*I5-II4-III6-V*	*0.497101738*	*I5-II4-III4-V*	*0.328591374*
I5-II9-III8-V	0.681220726	I5-II4-III3-V	0.30404971	*I5-II4-III9-V*	*0.515812781*	*I5-II4-III6-V*	*0.328514423*

Note that the Euclidean distance itself employs the square of the correlations factors, *i.e.*, a higher order statistical framework, which nevertheless may be further enriched with other statistical outputs and factors, although all directly or indirectly depend on the correlation factor [155].

In is also worth remarking that in the present uracil-derivative anti-HIV analysis, the four-descriptors’ dependency is not necessary in equation (17) since it is not needed in assessing the structural/reactivity parameters hierarchy in the minimum variational path principle of (16) by being absorbed in the rest of correlations by means of the *transitivity chain rule*:

- whenever two descriptors are common for adjacent activities’ correlations—they will be considered as a single common influence in chemical causes for the observed biological activity.

This way, the redundancies or double counting of models are avoided, even at the cost of “jumping” some intermediate models, like the four-descriptors’ endpoints. The results are displayed in Table 5. They are interpreted in the sense of establishing the minimum of three path hierarchies, and then compared at the global level; note that more than three minimum paths will produce redundant information. Accordingly, the minimum paths, for LoSMoC and BraS cases (i)/(ii) separately, are:

- For case LoSMoC/V2/(i):
(18) (α): I2-II7-III8-V δ[α]=0.029043119
(β): I2-II1-III8-V δ[β]=0.029516257
(γ): I2-II7-III7-V δ[γ]=0.029877668
- For case LoSMoC/V6/(ii):
(19) (α): I1-II4-III1-V δ[α]=0.12810286
(β): I1-II4-III2-V δ[β]=0.1281234
(γ): I1-II4-III3-V δ[γ]=0.12819186
- For case BraS/V5/(i):
(20) (α): I1-II2-III9-V δ[α]=0.301780663
(β): I1-II1-III9-V δ[β]=0.323160465
(γ): I1-II1-III6-V δ[γ]=0.328852605
- For case BraS/V2/(ii):
(21) (α): I2-II2-III6-V δ[α]=0.17028659
(β): I2-II2-III3-V δ[β]=0.170615371
(γ): I2-II2-III4-V δ[γ]=0.172894351

The variational results of Table 5 summarized by equations (19)–(21) are most involved in ensuring the reliability of the present approach because:

- All the LoSMoC least path lengths are shorter than those of BraS, this way confirming that the chain based SMILES intermediates are *prior* to those displaying branching SMILES conformations, *i.e.*, in accordance with the steps [A] →[B] of Figure 3 in pyrimidine-related uracil attack onreserve transcriptase;
- While passing from LoSMoC to BraS configurations in the chemical-biological interaction of uracil derivatives–reverse transcriptase binding phenomenology one notes the maintenance of the same criteria variants, namely V2 of Table 3:
(22) LoSMoC/V2/(i) → BraS/V2/(ii)
  meaning that the chain-to-branching passage seems to require the same features of the principal chain and of the secondary branch alike;
- Looking now to the cases interchanged in the transformation of equation (22) one also notes that the passage from case (i) based on longest chain in the SMILES configuration to the case (ii) based on the pyrimidinic N3 atom’s neighbors, happens consistently. The mechanism of interaction is described as involving the trans-membrane transduction by means of the longest chain of SMILES configuration; it is followed by the bonding stage centered on the N3 atom of the pyrimidine ring nuclei as already proved to be specific for spirodiazine derivatives in their transformations towards recorded anti-inflammatory activities, anti-HIV activity included [126].

With these we exposed the pre-final stage of ligand-receptor interaction explained by variational/spectral-QSAR analysis. It assumes the linking of the LoSMoC and BraS least paths’ models to mirror the successive SMILES transformations of the free molecule inside the HIV cell, by passing its lipidic walls and plasmidic environment hitting the reverse transcriptase palm-p66 pocket, see Figure 3E. 

### 2.5. OECD-QSAR Principle 5: A Mechanistic Interpretation

The intent of OECD QSAR Principle 5 is not to reject models that have no apparent mechanistic basis, but to ensure that some consideration is given to the possibility of a mechanistic association between the descriptors used in a model and the endpoint being predicted and to ensure that this association is documented. Since the physicochemical QSAR parameters were chosen in this study, a mechanistic interpretation of the models is possible. This nevertheless follows specific steps integrated in the previously discussed OECD-QSAR principles.

Accordingly, on the concrete study of actual uracils’ anti-HIV action, the transformation (22) is projected on the structural or chemical reactivity descriptors it encompasses for the shortest path lengths so that it concludes the variational QSAR modeling:

α_LoSMoC/V2/(i)_ → α_BraS/V2/(ii)_ (23)

which is equivalently rewritten with the help of Equations (18) and (21):

[I2-II7-III8-V] → [I2-II2-III6-V] (24)

and even more with the help of endpoint identifications of Table 4, respectively as:

[(χ)→(χ,ω)→(χ,ω,η)→(χ,ω,η,π,logP)] → [(χ)→(η,logP)→(logP,π, ω)→(χ,η,logP,π,ω)] (25)

Now, the solution of the structural/reactivity causes driving the ligand receptor binding mechanism in the present 1,3-disubstituted uracils against human immunodeficiency virus (HIV-1) action is given by combining the two variational principles noted before:

- Transitivity chain rule, and
- Minimization of redundancies

in structural/chemical reactivity dependencies. As such, the first alpha spectral-SAR hierarchy in (25) solves the first three causes:

χ→ω→η→(π,logP) (26a)

while the second alpha path hierarchy of (25) solves the last degeneracy of (26a) as explained next: one considers the already solved structural/reactivity causes of (26a) from where it results that η follows χ; with this ordering back in (25) one yields that logP follows η; this should finally applied also in (26a); all in all, the ordered causes of structural/reactivity influences in actual anti-HIV mechanism look like:

χ→ω→η→logP→π (26b)

**Figure 4:**
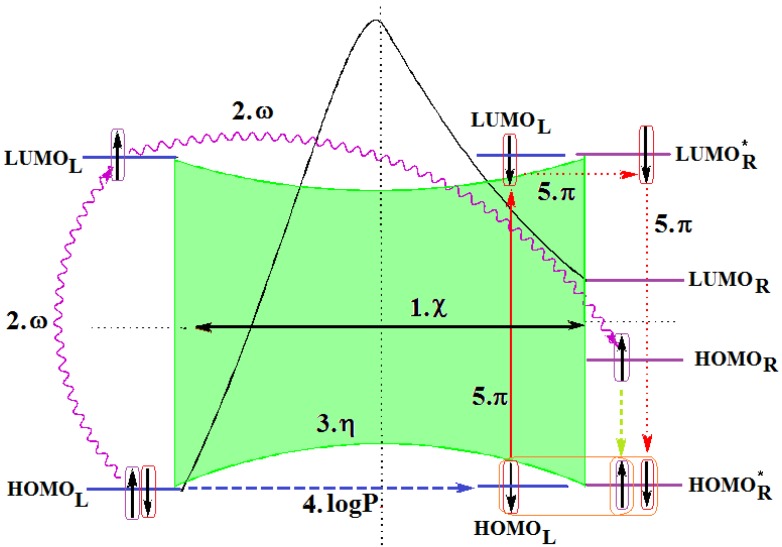
Representation of mechanistic molecular orbital interaction and bonding between the the uracil derivative compounds and HIV through binding the SMILES (essentially the BraS) molecule (the ligand, L) with the molecular pocket of the receptor (R) site, see the stages (**D**) & (**E**) of Figure 3, through variational principles of chemical reactivity of **1.** electronegativity (χ), **2.** electrophilicity (ω), **3.** chemical hardness (η), **4.** lipophilicity (logP) and **5.** chemical power (π), according with the Spectral-QSAR analysis of equations (16)–(26).

Equation (26b) may be represented by the orbital based scheme of chemical reactivity driving biological (anti-HIV) activity as provided in Figure 4. It is explained in the light of chemical reactivity principles, (see Section 2.2) within the “time-space” framework fixed by the chemical reactivity-biological activity interaction:

- The development time is not the physical one but an internal one related with the reaction coordinates, so that the reactivity-driven-activity steps are phenomenological ordered through being interrelated and inter-conditioned during the entire physical time of the binding (on a nano-second scale);
- The described interaction is spatially placed between the ligand (L) represented by the SMILES branched molecule resulted upon the HIV cell’s transduction (at least of the viral envelope) and the receptor–the palm region of the p66 region of the reverse transcriptase.

In these conditions the found mechanism for uracil derivatives’ anti-HIV activity goes as follows:

- The first step is triggered by electronegativity (χ) and of its principle of minimization difference between ligand (L) and receptor (R) HOMO-LUMO middle-levels, as provided by equation (3). In this stage the ligand and receptor are energetically aligned around a common electronegativity; it also associates with “preparation” of HOMO and LUMO states for ceding and accepting electrons by the accompanying interchanging charge;
- The second step accompanies the first one through the electrophilicity (ω) by putting into action the charge transfer by tunneling of the L-R barrier for one electron of the HOMO_L_ level passing to the LUMO_L_ and then down to the HOMO_R_ state by means of the LLR mechanism, see Figure 2b; the minimization principle for electrophilicity, equation (11), further allows the relaxation of the transferred electron from the HOMO_R_ to the HOMO_R_* level;
- The third step appears naturally “called” by the second one: the R to R* actually corresponds with the expansion of the HOMO_R_-LUMO_R_ gap to HOMO_R_*-LUMO_R_* to be equal with HOMO_L_-LUMO_L_ one, in accordance with the maximum hardness principle, equation (6), being this step driven by chemical hardness;

- The fourth step converts spatially the energetic HOMO-LUMO coupling of ligand-receptor by hydrophobicity/lipophilicity (logP) action eventually assuring also the capsid penetration; note that the previous charge transfer was realized through (quantum) tunneling, in accordance with electrophilicity driving action, thus being consistent with the earlier (second step) long-range action of the pyrimidines in the plasmidic region of HIV cell against its reverse transcriptase enzyme inside of the capsid, see Figure 3;
- The fifth and the last step is accomplished by chemical power (π) which assures the effective ligand-receptor binding (now also spatial in nature) by transferring the remaining electron of HOMO_L_ to LUMO_L_ and then to LUMO_R_* by means of the LRR mechanisms of Figure 1b; it nevertheless fulfils the minimization principle, equation (9), by undergoing the final LUMO_R_* to HOMO_R_* relaxation, when it pairs with the electron arrived from the electrophilicity step above.

Overall, the presented molecular mechanism fully explains the ligand-receptor binding in all respects:

- Spatially (the molecule is placed in the pocket of HIV’s reverse transcriptase);
- Energetically (all transitions compensate each other);
- By electronic pairing (assured by electrophilicity and chemical power actions);
- By bonding on the relaxed HOMO_R_* level

This way the presented variational QSAR anti-HIV mechanism assures the stabilization of pyrimidine complex with the enzyme transcriptase receptor towards the concerned apoptosis of the HIV cells through inhibiting his enzyme activities for further actions (and replications) in the host organism. This study complements the previous one [64], by effectively employing the various forms of SMILES configuration for the ligand molecules, with the satisfactory result that the proposed molecular anti-HIV mechanism appears to be reliable and self-consistent, aiding us to envisage ligand-receptor binding. However, while being aware of the importance the branching SMILES procedure has played in the actual endeavor, further study may be directed towards employing the topological branching information of the involved molecules, being this field equally rich and promising in QSAR chemical systems with high complexity [156,157,158,159]. Moreover, when the actual mechanistic analysis is envisaged to be further used in drug design, *i.e.*, in searching for new anti-HIV agents, one should employ the resulting minimum path, namely the path (α) in Equation (18), and the intermediate QSAR models contained along this path, *i.e.*, A(χ), A(χ,ω), and A(χ,ω,η), respectively, to further identify uracil derivative shapes best fulfilling the synergistic needs of all these models, finally tested also for external robustness. This step is under our purview in achieving the self-consistent *mechanistic drug design* in an *in-cerebro-in silico* framework. 

## 3. Conclusions

Chemical bonding and reactivity were at the forefront of modern chemistry in the last century, described through various qualitative theories (*viz*. Lewis’ theory of atoms and molecules [160] or the resonance theory of Pauling [161,162,163,164]) as well as through quantitative ones (e.g., Heitler-London homopolar theory [165], Hückel and extended Hückel heteropolar theories [166,167,168], or the Bader-Gillespie Atoms in Molecules–AIM [169,170,171] and Valence Shell Electron Pair Repulsion—VSEPR formulations [172,173], just to name a few), before finally being united within the *conceptual* Density Functional Theory [174,175] leading to the the recent bonding-by-reactivity scenario within the so- called chemical orthogonal space [54,55] of electronegativity [95] and chemical hardness [105]. The next step was made when chemical-biology binding interactions and binding were considered as a superior phenomenological level of ordinary chemical bonding. To treat it, however, the descriptors’ orthogonality feature turns out to be of prime importance so that the quantitative structure-activity relationship QSAR approach, while incorporating it, establishes itself as the current paradigm in modeling biological activity. Eventually it may fully employ the fundamental chemical reactivity concepts such as the electronegativity and chemical hardness along their second generation of descriptors such as chemical power [59,64] and electrophilicity [120], and their associated variational principles, while assuming a given (parabolic) electronic total energy *vs.* number of electrons *E = E(N)* shape dependency [117,176,177].

In this chemical reactivity-driven biological activity context, the present work has succeeded in clarifying the mechanism of molecular-cellular action by means of chemical reactivity indices and of their variational principles viewed as descriptors in a QSAR context, while studying available uracil derivatives’ anti-HIV action. 

This way, one is left with the variational QSAR recipe generally summarized following the Organization for Economic Co-Operation and Development (OECD) related principles (see Introduction):

- For QSAR-OECD Principle 1 (a defined endpoint): considering SMILES longest chain (LoSMoC)- and branching (BraS)-based counterparts of envisaged molecules as the actual molecular ansatz for modeling the envisaged anti-HIV activity by the end-point of half maximal effective concentration (EC_50_, μM) antiviral activity of 1,3-disubstituted uracils against human immunodeficiency virus (HIV-1)—see Table 1;
- For QSAR-OECD Principle 2 (an unambiguous algorithm): implementing QSAR orthogonal descriptors with associate min-max principles of chemical reactivity: electronegativity and chemical hardness, and of their mixed forms under electrophilicity and chemical power indices; the first two descriptors were also considered with “branching” working forms for BraS molecules up to the third order in HOMO and LUMO, within Koopmans theorem and spectral like resolution frameworks; the last two descriptors are merely associated with chemical charge transfer at the molecular frontier (HOMO and LUMO). Together, they all assure the *chemical reactivity-driving-biological activity* and provide the molecular mechanism linking structural causes with recorded biological effects (anti-HIV in the present application), while being accompanied by the hydrophobicity/lipophilicity index (logP) modeling the transduction through cellular HIV membranes;
- For QSAR-OECD Principle 3 (a defined domain of applicability): selecting the appropriate QSAR correlation through the screening based on chain (LoSMoC) and branching (BraS) SMILES molecular structures; this stage allows further application of transitivity and minimum redundancy rules for the QSAR descriptors as they are present in the various multi-linear computed endpoints;
- For QSAR-OECD Principle 4 (appropriate measures of goodness-of–fit, robustness and predictivity): ordering the multi-descriptor dependencies with the help of spectral-path length hierarchy for chain (LoSMoC) and branching (BraS) SMILES molecular interaction, and globally in between them, with the aim of Euclidian path measure and of their systematic minimum search across all QSAR models and of their combinations;
- For QSAR-OECD Principle 5 (a mechanistic interpretation, if possible): constructing the molecular (orbital/frontier) diagram describing the mechanism of ligand-receptor interaction based on correlating the least alpha paths of LoSMoC and BraS QSAR analyses with the chemical reactivity descriptors’ electronic manifestations and principles.

All these steps and algorithm were applied and directed for establishing the general molecular mechanism whereby 1,3-disubstituted uracils act against human immunodeficiency virus (HIV-1) by inhibiting its reverse transcriptase enzyme by means of ligand-receptor binding. Results were satisfactory and show reliability in all steps, while complementing the recent work where the resulting ligand-complex ended in an activated state [64], with the actual fully predicted bonding behavior; however, for future works, it would be interesting to research the biological effect of a mixture between a marine drug and a pyrimidine derivate with anti-HIV activity, as well as extending the branching study from SMILES to topological characterization of molecules aiming to identifying the best molecular shape responding to the best/minimal path in providing the ligand-receptor interaction and of its mechanism by the synergetic mechanistic drug design.

## Supplementary Materials

Supplementary materials can be accessed at: http://www.mdpi.com/1420-3049/18/8/9061/s1.
