# Supplementary material for: Determining Chemical Reactivity Driving Biological Activity from SMILES Transformations: The Bonding Mechanism of Anti-HIV Pyrimidines
Source: Molecules. 2013 Jul 30;18(8):9061–116. doi: 10.3390/molecules18089061 (PMC6270382; doi:10.3390/molecules18089061)
Supplement: Supplementary file 1 [file molecules-18-09061-s001.pdf]

Supplementary Material

Table S1. QSAR equations, whose correlations factors are reported in Table 4, obtained for cases V2/(i) and V6/(ii) for longest SMILES molecular chain (LoSMoC) and respectively for the cases V5/(i) and V2/(ii) for branching SMILES (BraS) selected compounds' sets form Table 3 with respective molecules of Table 1.

No.	A(x)	LoSMoC		*BraS*	
		**Case V2/(i)**	**Case V6/(ii)**	***Case V5/(i)***	***Case V2/(ii)***
$\mathbf{I_1}$	A(logP)	4.26912+0.55125logP	4.23355+0.81494logP	*4.84491+0.58027logP*	*4.52274+0.71566logP*
$\mathbf{I_2}$	A(χ)	22.67275-0.66377χ	4.55124+0.03175χ	*10.93556-0.39938χ*	*15.11277-0.66651 χ*
$\mathbf{I_3}$	A(η)	7.08567-1.69991η	7.03106-1.30862η	*7.4902-1.06418η*	*4.78306+0.32872η*
$\mathbf{I_4}$	A(π)	3.92170+0.09456π	4.29847+0.10588π	*3.61236+0.42869π*	*7.84605-0.73058 π*
$\mathbf{I_5}$	A(ω)	4.58879+0.00133ω	4.55497+0.00328ω	*4.34061+0.01424ω*	*8.60928-0.06774ω*
$\mathbf{II_1}$	A(logP, χ)	21.14033+0.24164logP-0.61809χ	11.76769+1.40005logP-0.35099χ	*10.2305+0.55523logP-0.36445χ*	*12.55396+0.39884logP-0.52832χ*
$\mathbf{II_2}$	A(logP, η)	6.39574+0.565559ogP-1.73893η	2.04347+1.22009logP+1.25565η	*7.30168+0.58186logP-1.06862η*	*-2.23842+1.41616logP+2.42946η*
$\mathbf{II_3}$	A(logP, π)	2.60849+0.64372logP+0.1365π	5.01171+1.34392logP-0.15525π	*3.2110+0.60397logP+0.48713π*	*8.72544+1.2314logP-1.58241π*
$\mathbf{II_4}$	A(logP, ω)	3.00965+0.64042logP+0.00382ω	4.86736+1.44907logP-0.00643ω	*3.9169+0.59713logP+0.0187ω*	*8.23622+0.96707logP-0.0906ω*
$\mathbf{II_5}$	A(χ, η)	24.68432-0.77517χ+0.7752η	14.20663-0.24218χ-2.40449η	*11.55233-0.30411χ-0.87949η*	*14.08942-0.68814χ+0.56381η*
$\mathbf{II_6}$	A(χ, π)	25.83622-0.74594χ-0.08635π	9.80107-0.28572χ+0.23827π	*9.75932-0.42984χ+0.48708π*	*15.77572-0.61145χ-0.46412π*
$\mathbf{II_7}$	A(χ, ω)	24.97069-0.71282χ-0.00331ω	11.942-0.37468χ+0.00975ω	*11.10894-0.51674χ+0.03159ω*	*14.47986-0.51569 χ-0.03396ω*
$\mathbf{II_8}$	A(η, π)	31.84472-11.64925η-1.10667π	15.22197-4.98754η-0.35134π	*17.06452-3.1773η-1.41281π*	*27.95051-4.768η-3.55261π*
$\mathbf{II_9}$	A(η, ω)	23.05772-7.58872η-0.02910ω	13.60228-4.45866η-0.01058ω	*12.99415-2.22135η-0.05765ω*	*16.80535-2.1844η-0.13506ω*
$\mathbf{II_{10}}$	A(π, ω)	6.32772+1.84517π-0.07405ω	3.0013+0.93443π-0.02879ω	*3.51483+3.04685π-0.17522ω*	*7.52033+2.46816π-0.2151ω*

Table S1. *Cont.*

No.	A(x)	LoSMoC		*BraS*	
		Case V2/(i)	Case V6/(ii)	*Case V5/(i)*	*Case V2/(ii)*
III_1	A(logP, χ, η)	22.82486+0.19436logP-0.70371χ+0.53363η	11.3077+1.44071logP-0.34311χ+0.16674η	*10.85633+0.56337logP-0.26567χ-0.90714η*	*4.31675+1.09484logP-0.36691χ+2.07817η*
III_2	A(logP, χ, π)	23.96141+0.16122logP-0.69332χ-0.06308π	11.60567+1.43274logP-0.33994χ-0.015π	*8.89935+0.57922logP-0.39662χ+0.53862π*	*9.91647+1.13856logP-0.09695χ-1.47594π*
III_3	A(logP, χ, ω)	23.32971+0.15983logP-0.66921χ-0.0024ω	11.65709+1.41233logP-0.34478χ-0.00022ω	*10.39432+0.57803logP-0.4935χ+0.03513ω*	*5.73251+1.14593logP+0.21387χ-0.10884ω*
III_4	A(logP, η, π)	35.60159-0.19751logP-13.04848η-1.26382π	11.28348+1.25599logP-2.88492η-0.40264π	*14.38421+0.53345logP-2.62796η-1.0428π*	*13.96946+1.0749logP-1.27018η-2.22594π*
III_5	A(logP, η, ω)	24.37105-0.11209logP-8.01479η-0.03124ω	10.06553+1.32589logP-2.57483η-0.01361ω	*11.75451+0.54133logP-2.00173η-0.0465ω*	*6.75247+1.05103logP+0.38681η-0.08067ω*
III_6	A(logP, π, ω)	5.99221+0.10487logP+1.76355π-0.07031ω	3.56242+1.3936logP+0.77764π-0.03275ω	*3.13909+0.5753logP+2.92716π-0.16349ω*	*8.60002+1.13343logP-0.97002π-0.03662ω*
III_7	A(χ, η, π)	30.62119-0.31409χ-7.28923η-0.73325π	15.52764-0.21645χ-3.22381η-0.08936π	*20.47952+0.29761χ-4.98913η-2.50334π*	*24.49257-0.24611χ-3.19096η-2.51196π*
III_8	A(χ, η, ω)	23.33922-0.06411χ-6.95115η-0.02696ω	14.26666-0.23567χ-2.49622η-0.0004ω	*14.60619+1.40346χ-7.35393η-0.27092ω*	*17.3936+0.25108χ-3.10298η-0.17982ω*
III_9	A(χ, π, ω)	25.07721-0.71689χ-0.01061π-0.00290ω	35.70574-1.34506χ-2.78601π+0.12215ω	*-164.29004+11.57445χ+62.83296π-4.28182ω*	*17.00378-0.70238χ-0.90355π+0.03221ω*
III_{10}	A(η, π, ω)	22.92367-7.52746η+0.01595π-0.02951ω	10.71363-3.31225η+0.33826 π-0.01862ω	*10.29369-1.59027η+0.893π-0.09276ω*	*21.36918-3.23757η-1.38771π-0.08466ω*
V	A(logP, χ, η, π, ω)	31.05835-0.11186logP-0.18488χ-8.86159η-0.69978π-0.00725ω	125.59791+1.39459logP-4.02309χ-10.40029η-12.22228π+0.45065ω	*-165.13829+0.57858logP+11.5865χ+0.06933η+62.86877π-4.27789ω*	*4.22748+1.11128logP+0.86326χ-1.70754η+1.20751π-0.27528ω*
